# Mild Silver-Mediated Geminal Difluorination of Styrenes Using an Air- and Moisture-Stable Fluoroiodane Reagent**

**DOI:** 10.1002/anie.201408812

**Published:** 2014-10-21

**Authors:** Nadia O Ilchenko, Boris O A Tasch, Kálmán J Szabó

**Affiliations:** Department of Organic Chemistry, Stockholm University

**Keywords:** fluorine, hypervalent compounds, reaction mechanism, rearrangement, silver

## Abstract

An air- and moisture-stable fluoroiodane in the presence of AgBF_4_ is suitable for selective geminal difluorination of styrenes under mild reaction conditions. One of the C=F bonds is formed by transfer of electrophilic fluorine from the hypervalent iodine reagent, while the other one arises from the tetrafluoroborate counterion of silver. Deuterium-isotope-labelling experiments and rearrangement of methyl styrene substrates suggest that the reaction proceeds through a phenonium ion intermediate.

Organofluorines are very important substances in pharmaceutical and agrochemical industries and have also had an increasing role in medicinal research and diagnostics.[1] As organofluorine compounds are specific regulator substances, inhibitors, or biomarkers, constant development of new methodologies for their selective synthesis is a very important task in synthetic chemistry. The appearance of new stable, electrophilic fluorinating reagents and methodologies allowed the extension of the new methodologies for selective synthesis of a large variety of organofluorine compounds.[2] In recent years creation of single C=F bonds, introduction of CF_2_, and trifluoromethylation reactions have received a lot of attention. Of these major areas the introduction of a CF_2_ group is probably the least developed.[3] Nevertheless, the difluoromethyl group proved to be an important motif in enzyme inhibitors.[4] This property is probably a result of the ability of the CF_2_H group to donate hydrogen bonds,[5] and, thus serve as a bioisoster for hydroxy and thiol moieties.[6] Furthermore, very recently CF_2_ groups have found new applications in medicinal diagnostics as well. A new trend in the synthesis of trifluoromethyl-group-based PET tracers is substitution of suitable sp^3^ difluoromethyl groups with ^18^F to obtain ^18^FCF_2_ functionalities.[7]

The early methods for introducing difluoromethyl groups to organic molecules were mainly based on the application of highly reactive inorganic fluorinating reagents, such as DAST,[8] Deoxofluor,[9] and XeF_2_.[10] However, application of these reagents may lead to problems in functional-group tolerance and selectivity, and causes hazardous HF development upon contact with water. The recently reported methodologies are usually based on metal-catalyzed cross-coupling of various CF_2_ carriers with aryl halides and boronates. For example the groups of Amii,[11] Hartwig,[12] Prakash and Olah,[13] and Qing[14] used a copper catalyst (or mediator), while the group of Zhang[15] used palladium catalysis for the introduction of a CF_2_ group to organic substrates. Baran and co-workers have published a series of papers on C=H difluoromethylation by CF_2_H radicals.[16] Introduction of the difluoromethyl group with consecutive difluorination reactions is a less common approach compared to the above-mentioned cross-coupling of CF_2_ units with the organic substrate. Tang and co-workers[17] have shown that consecutive geminal difluorination of aromatic benzyl groups can be achieved by using Selectfluor as a fluorine source in combination with a silver catalyst under oxidative conditions. A similar reaction has been reported by Chen and co-workers[18] using visible-light-promoted reactions under metal-free conditions. For both reactions a radical mechanism was postulated.

Recently, development of new fluorination reactions by application of hypervalent iodine reagents has attracted considerable attention.[2a,b,^19^] As a part of our synthetic fluorochemistry program,[20] we have studied the potential use of the hypervalent fluoroiodane **1**[19f,^21^] as an electrophilic organofluorinating reagent [see Eq. (1)]. The fluoroiodane **1** is an air- and moisture-stable crystalline compound and structural analogue of the Togni reagent,[2a,^22^] which has been one of the most successful electrophilic trifluoromethylating reagents in organic synthesis. Another attractive property is that **1** can be obtained from its chloro analogue by addition of KF.[21b] Thus, synthesis of **1** using KF involves a simple umpolung method by changing a nucleophilic fluorine into an electrophilic one. This simple possibility for umpolung of the fluorine atom can be a useful feature for the development of new tracers for medicinal diagnostics.[23] The first report by Legault and Prévost[21a] on the attempts to use **1** for C=F bond-formation reactions was disappointing. In contrast to its bromo analogue, **1** was not suitable for the electrophilic halogenation of anisole. According to Stuart and co-workers[19f] **1** reacts with 1,3-diketoesters and 1,3-diketones in the presence of TREAT-HF to give mono- and difluoro products, respectively. As far as we know, this paper by Stuart and co-workers[19f] has been the only report on the application of **1** for creating C=F bonds.

We have now found [Eq. (1)] that mixing of equimolar amounts of the styrene **2 a**, **1**, and AgBF_4_ (**3**) results in the difluoro compound **4 a** under mild reaction conditions (40 °C). The reaction was very clean and the yield of the isolated product was over 50 %, thus indicating that in the geminally difluorinated product one of the fluorine atoms was derived from **1**, while the other one originated from the BF_4_^−^ counterion of silver. Thus, under mild reaction conditions, using air- and moisture-stable starting materials a formal F_2_ addition to **2 a** could be performed.

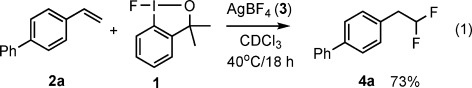
(1)

Deviation from the above mentioned [Eq. (1)] optimal reaction conditions led to either lower yields of isolated **4 a** or no reaction of **2 a** (Table 1). Application of catalytic amounts of AgBF_4_, instead of 1 equivalent, led to a significant drop in yield from 73 to 36 % (entry 1). Using AgSbF_6_ as a mediator and F source led to formation of **4 a** (entry 2) but the reaction was much slower than that with AgBF_4_, as a lot of unreacted starting material (**2 a**) remained. When AgOAc, AgCN, or AgF was applied instead of **3** the styrene **2 a** remained intact (entry 3). A combination of catalytic amounts of AgOAc and 1 equivalent Bu_4_NBF_4_ was inefficient (entry 4), but upon treatment with (*t*Bu)_3_PHBF_4_, instead of Bu_4_NBF_4_, **4 a** was isolated with 44 % yield (entry 5). This result and the above experiment (entry 1) with a catalytic amount of **3** indicate that the geminal difluorination reaction can be achieved with a catalytic amount of a silver salt. Thus, the source of the second fluoride [Eq. (1)] is the BF_4_^−^, which must not necessarily be added as part of a silver salt. However, the yields of **4** are much higher with stoichiometric amounts of AgBF_4_. Therefore, we employed one equivalent of AgBF_4_ as a mediator and secondary fluorine source in additional applications (see also Table 2). When we replaced **1** with Selectfluor, fluorination of **2 a** was not observed (entry 6). In the case of using Zn(BF_4_)_2_ or Cu(MeCN)_4_BF_4_, instead of AgBF_4_, we could observe the difluorination reaction and isolate **4 a** in the corresponding yields of 46 and 32 %. However, particularly with a copper salt, several other byproducts were formed (entries 7 and 8).

**Table 1:** Variation of the yield of the isolated product resulting from changes to the reaction conditions [see Eq. (1)].

Entry	Deviation from the reaction conditions given in Equation (1)	Yield [%]
1	10 mol % AgBF_4_	36
2	1 equiv AgSbF_6_	18
3	1 equiv AgOAc, AgCN and AgF	<5
4	10 mol % AgOAc, 1 equiv Bu_4_NBF_4_	<5
5	10 mol % AgOAc, 1 equiv (*t*Bu)_3_PHBF_4_	44
6	1 equiv AgBF_4_, 1 equiv Selectfluor instead of **1**	<5
7	1 equiv Zn(BF_4_)_2_	46
8	1 equiv Cu(MeCN)_4_BF_4_	32
9	1 equiv ZnF_2_, CuF_2_	<5
10	MeOH as solvent	<5
11	toluene as solvent	trace

The reaction with palladium salts was rather interesting. [Pd(MeCN)_4_(BF_4_)_2_] proved to be a rather efficient catalyst, thus affording **4 a** with 50 % yield [Eq. (2)]. However, when the BF_4_^−^ counter ion exchanged to Cl^−^ the outcome of the reaction was completely different. When using [PdCl_2_(MeCN)_2_] as the catalyst **4 a** did not form at all but the reaction gave the iodofluorinated product **5**, which could be isolated with 43 % yield. Further studies indicated that in the geminal difluorination reaction with **1**, AgF, ZnF_2_, or CuF_2_ were not able to serve as fluoride sources (Table 1, entries 3 and 9). We employed CDCl_3_ as the solvent, which allowed the careful analysis of the crude reaction mixtures by ^1^H and ^19^F NMR spectroscopy. Change of the solvent to MeOH or THF completely hindered any transformation of **2 a** (entry 10), while using toluene led to formation of only traces of **4 a** (entry 11).

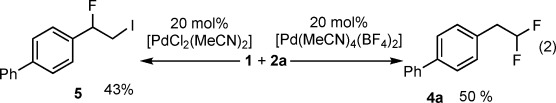
(2)

Subsequently, we studied the synthetic scope of the reaction (Table 2). Both α- and β-naphtyl styrenes (**2 b** and **2 c**) underwent geminal difluorination with high yields (entries 2 and 3). Despite the use of stoichiometric amounts of AgBF_4_ the bromo substituent in **2 d** was tolerated and **4 d** was formed in a clean difluorination process (entry 4). The compound **4 d** can be a useful intermediate for modular synthesis of difluoromethyl compounds, as the bromo functionality can easily transformed by Suzuki–Miyaura coupling.[24] The difluorination reaction also tolerates oxygen-containing substituents, such as the ether in **2 e** and carboxylate in **4 f** (entries 5 and 6). However, for these substrates, we obtained somewhat lower yields than those for the hydrocarbon substrates **2 a**–**c**. The parent styrene **2 g** reacted readily with **1** but the isolation of **4 g** was difficult because of its volatility. Therefore, we determined the yield of **4 g** by ^1^H NMR spectroscopy. *meta*-Phenyl (**2 h**) and bromo (**2 i**) styrenes reacted readily to provide the corresponding difluorinated compounds **4 h** and **4 i** (entries 8 and 9). However, our attempts to obtain geminal difluorinated products from *ortho*-substituted styrenes remained fruitless. The reactions with the α-methyl styrenes **2 j**–**l** gave surprising results. We still obtained the β-difluorinated products **4 j**–**l** but the methyl group migrated from the α- to the β-position (entries 10–12). The rearrangement proceeds very cleanly, as we could not observe any other isomers of **4 j**–**l** in the crude reaction mixture.

**Table 2:** Difluorination of styrene derivatives with the iodiane 1 and AgBF_4_.^[a]^

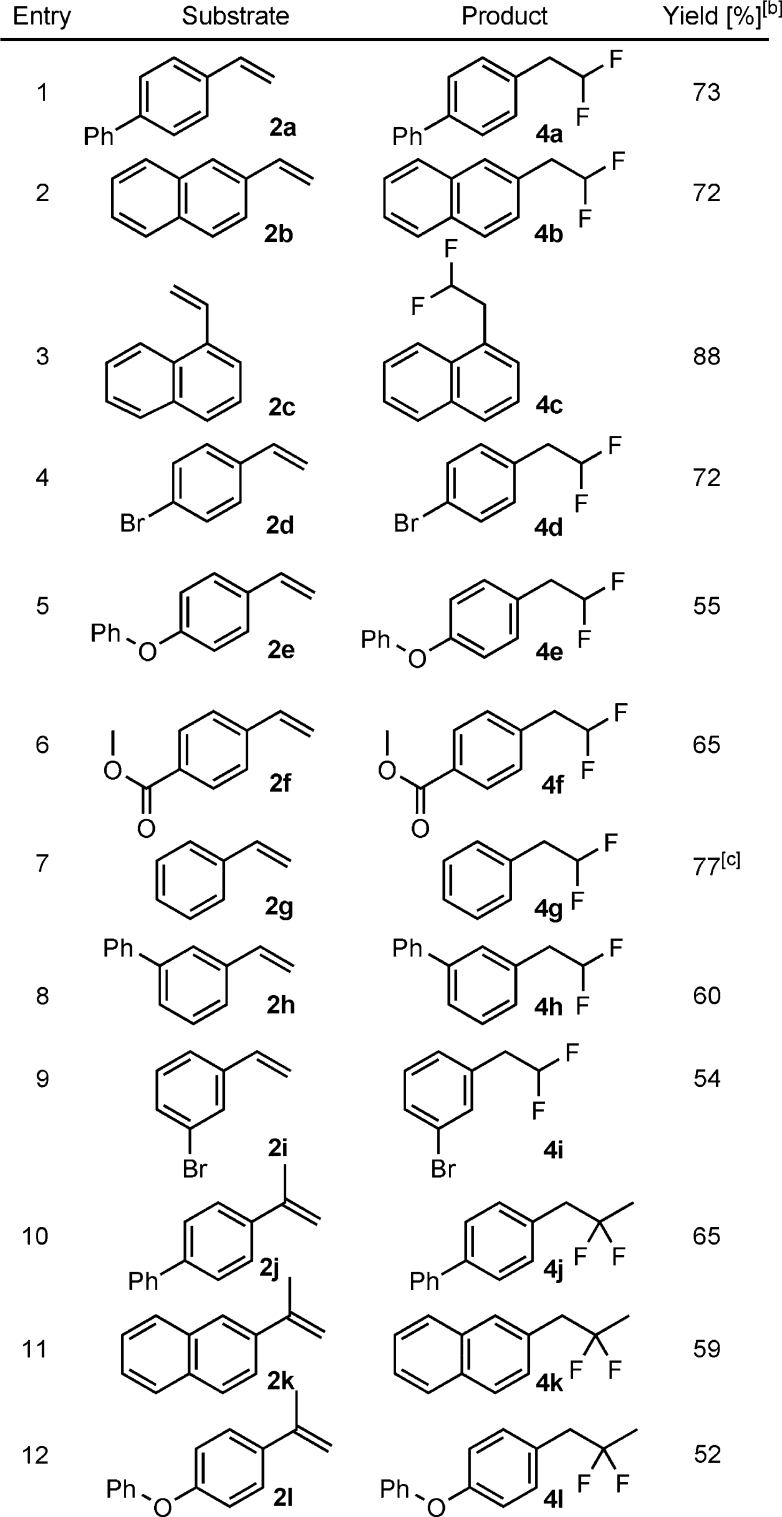

[a] Styrene **2** (0.1 mmol), **1** (0.1 mmol), and AgBF_4_ (0.1 mmol) in chloroform (0.5 mL) was stirred at 40 °C for 18 h. [b] Unless otherwise stated yield is that of isolated product. [c] Yield determined by NMR spectroscopy.

This finding and the fact that one of the fluorines arises from an electrophilic source (from **1**) and the other from a nucleophilic reagent (from a BF_4_^−^ anion) suggests a very interesting mechanism for the geminal difluorination process. To explore the mechanism, we prepared the deutero analogue of **2 a**, [D_2_]-**2 a**. When [D_2_]-**2 a** was reacted with **1** in the presence of AgBF_4_ under the usual reaction conditions the compound [D_2_]-**4 a** was obtained in a very clean reaction [Eq. (3)]. This outcome was a surprising result, since the shift of one of the deuterium atoms of [D_2_]-**2 a** was expected. The double deuterium shift suggested a significant deuterium isotope effect. Therefore, we performed a competitive difluorination reaction between **2 a** and [D_2_]-**2 a** [Eq. (4)]. However, according to these studies the deuterium isotope effect was found to be weak (about 1.3).

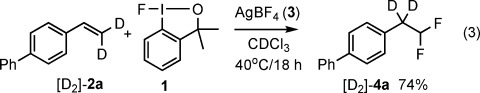
(3)

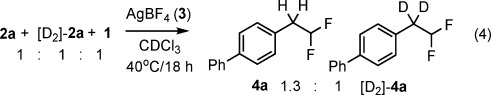
(4)

The above results indicate that the hydrogen shift from the α- to the β-position of styrene is unlikely. We reasoned that the difluorination reaction probably proceeds by an α- to β-carbon atom exchange of the styrene derivative through a possible phenonium ion intermediate. Such types of intermediates were first suggested by Cram[25] and subsequently observed by Olah and co-workers.[26]

Accordingly, our plausible mechanism (Figure 1) involves silver-activated addition of the iodane to the double bond of [D_2_]-**2 a** to give the iodonium ion **6**. Togni and co-workers[27] studied the activation of the CF_3_ analogue of **1** using zinc salts. A similar metal-mediated activation is conceivable for **1** as well. As mentioned above (Table 1, entry 7) Zn(BF_4_)_2_ is also a viable mediator of the difluorination process. Moreover, suitable copper and palladium salts [Table 1, entry 8 and Eq. (2)] may have a similar activating effect. The electron deficiency of iodine in **6** can be relieved by fluorine migration to give **7**. The π donation of the aromatic ring in **7** may result in formation of the phenonium ion **8**. In this process the iodoaryl group arising from **1** serves as a leaving group. The positive charge may delocalize over five carbon atoms (for sake of clarity only one of the resonance structures is given in Figure 1).

**Figure 1:**
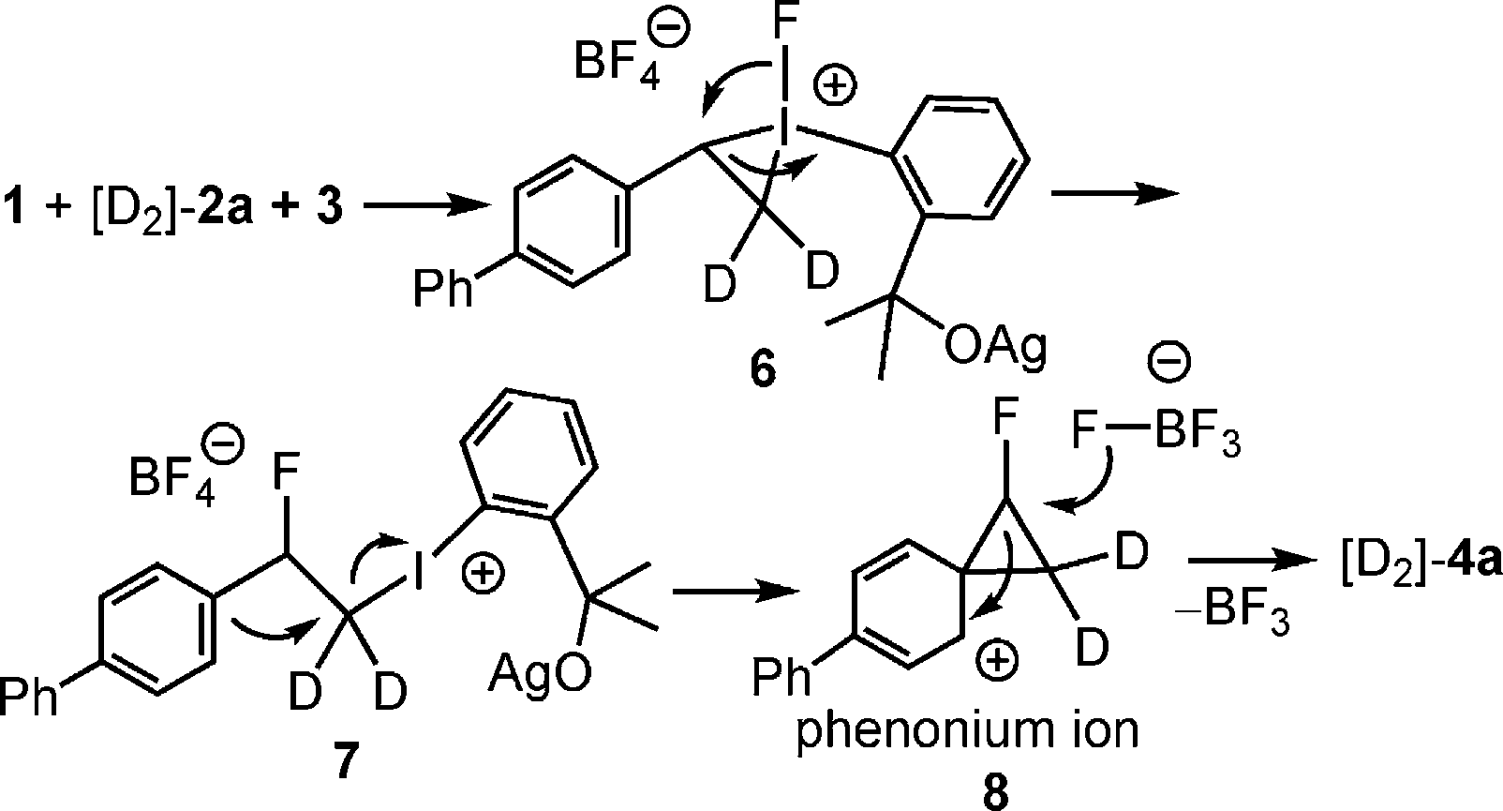
Plausible mechanism for the geminal difluorination of styrenes.

By using the [PdCl_2_(MeCN)_2_] catalyst [Eq. (2)] the faith of **7** was probably different and it underwent C(sp^2^)=I bond fission to result in **5**. The only possible source of iodine in **5** is **1**. However, in the case of AgBF_4_ (or a few other catalysts or mediators) the fluorinated phenonium ion **8** forms (Figure 1), and may undergo a second fluorination by a nucleophilic fluorine arising from the BF_4_^−^. The BF_4_^−^ anion is an unexpected but not unusual source of nucleophilic fluoride. Our recent studies[28] have shown the BF_4_^−^ from [Pd(MeCN)_4_(BF_4_)_2_] [see Eq. (2)] may serve as a fluorine source in the activation of (SiMe_3_)_2_ for catalytic silylation of allyl alcohols. Gandon and co-workers[29] have recently shown that BF_4_^−^ from AgBF_4_ is an excellent fluorinating reagent for organometallic compounds. The present study is probably one of the few examples demonstrating that a C=F bond can also be formed under mild reaction conditions using BF_4_^−^ as a fluorine source. An additional remarkable feature of the above-presented reaction is that the opening of the cyclopropyl ring of **8** is highly regioselective. We could not observe formation of α-,β-difluorinated regioisomers of **4 a** in the ^1^H and ^19^F NMR spectra of the crude reaction mixtures. Opening of the cyclopropane ring of the fluorinated carbon in **8** also easily explains the apparent migration of the methyl group in the difluorination of **2 j**–**l** (entries 10–12 in Table 2). A further confirmation of the plausible mechanism in Figure 1 could be the analogue reactivity of phenyl iodonium acetate (PIDA) or phenyl iodonium trifluoroacetate (PIFA) with styrene derivatives. PIDA and PIFA can be considered structural analogues of **1**. Tellitu and Domínguez,[30] and the group of Wirth[31] also postulated that the dioxo substitution of styrenes with PIDA and PIFA probably proceeds via phenonium intermediates.

In summary, we have shown that the hypervalent iodine **1** and AgBF_4_ (and a few other metal tetrafluoroborates) induce geminal difluorination of styrenes. These air- and moisture-stable reagents react under mild reaction conditions to perform a selective formal F_2_ addition. Using equimolecular amounts of **1** and styrene derivatives resulted in yields over 50 %, thus indicating that one of the C=F bonds is created by electrophilic fluorinating reagent **1**, while the other one results from fluorine transfer from BF_4_^−^. The deuterium-labelling experiments indicate that the process probably proceeds via phenonium intermediates. The reaction is suitable for mild synthesis of β-difluoro aromatic compounds, which are bioisosters[4–6] of natural compounds with benzylalcohol and thiol motifs. In addition, our method extends the scope of the fluorination reactions, including a new application of the easily accessible, stable, and safe electrophilic fluoroidane reagent **1**.

## Experimental Section

The iodane **1** (28.0 mg, 0.1 mmol), AgBF_4_ **3** (19.4 mg, 0.1 mmol), and the styrene **2** (0.1 mmol, 1 equiv) were mixed in CDCl_3_ (0.5 mL) and this mixture was stirred at 40 °C for 18 h. Then product **4** was isolated by chromatography.
