# Supplementary material for: Mild Silver-Mediated Geminal Difluorination of Styrenes Using an Air- and Moisture-Stable Fluoroiodane Reagent
Source: Angew Chem Int Ed Engl. 2014 Oct 21;53(47):12897–901. doi: 10.1002/anie.201408812 (PMC4501311; doi:10.1002/anie.201408812)
Supplement: Supplementary file 1 [file anie0053-12897-sd1.pdf]

Angewandte Chemie
Eine Zeitschrift der Gesellschaft Deutscher Chemiker

Supporting Information

© Wiley-VCH 2014

69451 Weinheim, Germany

Mild Silver-Mediated Geminal Difluorination of Styrenes using an Air- and Moisture-Stable Fluoroiodane Reagent**

*Nadia O. Ilchenko, Boris O. A. Tasch, and Kálmán J. Szabó**

anie_201408812_sm_miscellaneous_information.pdf

Supporting Information

Contents:

General Information 2

Experimental Procedures and Spectral Data 2

Competitive difluorination 6

References 7

NMR spectra of products 2a-d$_2$ and 4a-l 8

General Information

Hypervalent iodine **1**[1] and styrenes[2] **2a-d$_2$**, **2h**, **2j**, **2k** and **2l** were prepared according to literature procedures. All other chemicals were obtained from commercial sources and used as received. ^{1}H NMR, ^{13}C NMR and ^{19}F NMR spectra were recorded in $CDCl_3$ (internal standard: 7.26 ppm, ^{1}H; 77.2 ppm, ^{13}C) using 400 MHz spectrometers. High resolution mass data (HRMS) were obtained using APCI technique except for compound **4f** for which ESI technique was used. For column chromatography, silica gel (35-70 microns) was used. Unless otherwise stated the reactions were conducted under Ar.

Experimental Procedures and Spectral Data

General procedure for difluorination of styrenes:

Iodane reagent **1** (28.0 mg, 0.1 mmol), the corresponding styrene **2** (0.1 mmol) $AgBF_4$ **3** (19.4 mg, 0.1 mmol) were mixed in $CDCl_3$ (0.5 ml). This reaction mixture was stirred at 40°C for 18 h. The products were isolated by silica gel column chromatography.

4-(2,2-dideuterovinyl)-1,1'-biphenyl (2a-d$_2$)

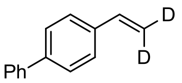

This compound was prepared according to the literature procedure[2] given for the non-deuterated analog (**2a**) using methyl-d^3-triphenylphosphonium bromide (95% deuterium) as starting material. The product was isolated as colorless solid using pentane as eluent system (341 mg, 93 % yield containing 87 % deuterium isotope according to NMR). ^{1}H-NMR (400 MHz, $CDCl_3$) δ 6.76 (s, 1H); 7.38-7.32 (m, 1H); 7.53-7.41 (m, 4H); 7.64-7.56 (m, 4H); ^{13}C-NMR (100 MHz, $CDCl_3$) δ 126.8; 127.1; 127.4; 127.5; 128.9; 136.4; 136.5 (d, J_{CD} = 2.2 Hz); 136.8; 140.7; 140.9; HRMS (APCI): *m/z* calcd. for $[C_{14}H_{10}D_2+H]^+$ 183.1137, found 183.1145; Mp: 122-125 ^{o}C.

4-(2,2-difluoroethyl)-1,1'-biphenyl (4a)

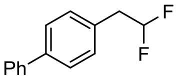

This compound was prepared according to the above general procedure. The product was isolated as a white solid using pentane:dichloromethane, 5:1 as eluent system (16 mg, 73 %). ^{1}H-NMR (400 MHz, $CDCl_3$) δ 3.19 (td, J_{HF} = 17.3, J = 4.6 Hz, 2H); 5.97 (tt, J_{HF} = 56.5, J =4.5 Hz, 1H); 7.32-7.37 (m, 3H); 7.42-7.47 (m, 2H); 7.55-7.59 (m, 4H); ^{19}F-NMR (376 MHz, $CDCl_3$) δ -114.75 (dt, J_{HF} = 56.4, 17.2 Hz); ^{13}C-NMR (100 MHz, $CDCl_3$) δ 40.7 (t, J_{CF} = 21.9 Hz); 116.7 (t, J_{CF} = 241.5 Hz); 127.2; 127.5; 127.6; 128.9;

130.4; 131.6 (t, J_{CF} = 5.9 Hz); 140.6; 140.8; HRMS (APCI): *m/z* calcd. for $[C_{14}H_{12}F_2]^+$ 218.0902, found 218.0899; Mp: 60-63 ^oC.

4-(1,1-difluoro-2,2-dideuteropropan-2-yl)-1,1'-biphenyl (4a-d$_2$)

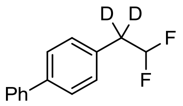

This compound was prepared according to the above general procedure starting from **2a**-d$_2$. The product was isolated as a white solid using pentane:dichloromethane, 5:1 as eluent system (16 mg, 74 % yield, containing 87 % deuterium). ^{1}H-NMR (400 MHz, $CDCl_3$) δ ^{1}H NMR (400 MHz $CDCl_3$) δ 5.96 (t, J_{HF} = 56.5 Hz, 1H); 7.39-7.32 (m, 3H); 7.48-7.42 (m, 2H); 7.61-7.55 (m, 4H); ^{19}F-NMR (376 MHz, $CDCl_3$) δ -114.97 - -115.17 (m); ^{13}C-NMR (100 MHz, $CDCl_3$) δ 29.9; 116.8 (t, J_{CF} = 241.5 Hz); 127.3; 127.6; 127.6; 129.0; 130.4; 131.5 (t, J_{CF} = 5.2 Hz); 140.7; 140.9. HRMS (APCI): *m/z* calcd. for $[C_{14}H_{10}D_2F_2]^+$ 220.1027, found 220.1024; Mp: 65-67 ^oC.

2-(2,2-difluoroethyl)naphthalene (4b)

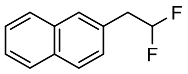

This compound was prepared according to the above general procedure. The product was isolated as white solid using pentane:dichloromethane, 5:1 as eluent system (14 mg, 72 %).^{1}H-NMR (400 MHz, $CDCl_3$) δ 3.34 (td, J_{HF} = 17.2, J = 4.6 Hz, 2H); 6.03 (tt, J_{HF} = 56.5, J = 4.6 Hz, 1H); 7.38-7.40 (m, 1H); 7.47-7.54 (m, 2H); 7.73 (s, 1H); 7.82-7.87 (m, 3H). ^{19}F-NMR (376 MHz, $CDCl_3$) δ -114.55 (dt, J_{HF} = 56.6, 17.2, Hz); ^{13}C-NMR (100 MHz, $CDCl_3$) δ 41.0 (t, J_{CF} = 21.9 Hz); 116.7 (t, J_{CF} = 241.6 Hz); 126.0; 126.3; 127.6; 127.7; 127.75; 128.4; 128.7; 129.9 (t, J_{CF} = 5.8 Hz); 132.6; 133.4; HRMS (APCI): *m/z* calcd. for $[C_{12}H_{10}F_2]^+$ 192.0745, found 192.0751; Mp: 64-67 ^oC.

1-(2,2-difluoroethyl)naphthalene (4c)

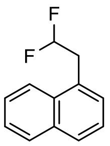

This compound was prepared according to the above general procedure. The product was isolated as colorless oil using pentane:dichloromethane, 5:1 as eluent system (17 mg, 88 %). ^{1}H-NMR (400 MHz, $CDCl_3$) δ 3.62 (td, J_{HF} = 16.6, J = 4.7 Hz, 2H); 6.09 (tt, J_{HF} = 57.0, J = 4.7 Hz, 1H); 7.42-7.59 (m, 4H); 7.81-7.90 (m, 2H); 7.99-8.01 (m, 1H). ^{19}F-NMR (376 MHz, $CDCl_3$) δ -113.38 (dt, J_{HF} = 56.7, 16.5 Hz); ^{13}C-NMR (100 MHz, $CDCl_3$) δ 38.0 (t, J_{CF} = 22.5 Hz); 116.6 (t, J_{CF} = 241.9 Hz); 123.4; 125.6; 126.0; 126.6; 128.5; 128.6; 128.9 (t, J_{CF} = 5.9 Hz); 129.1; 132.3; 134.0; HRMS (APCI): *m/z* calcd. for $[C_{12}H_{10}F_2+H]^+$ 193.0823, found 193.0817.

1-Bromo-4-(2,2-difluoroethyl)benzene (4d)

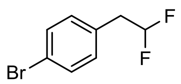

This compound was prepared according to the above general procedure. The product was isolated as colorless oil using pentane as eluent system (16 mg, 72 %). ^{1}H-NMR (400 MHz, CDCl$_3$) δ 3.10 (td, J_{HF} = 17.3, J = 4.4 Hz, 2H); 5.90 (tt, J_{HF} = 56.5, J = 4.5 Hz, 1H); 7.10-7.14 (m, 2H); 7.44-7.48 (m, 2H); ^{19}F-NMR (376 MHz, CDCl$_3$) δ -115.15 (dt, J_{HF} = 56.4, 17.3 Hz); ^{13}C-NMR (100 MHz, CDCl$_3$) δ 40.5 (t, J_{CF} = 22.1 Hz); 116.1 (t, J_{CF} = 241.6 Hz); 121.7; 131.3 (t, J_{CF} = 5.8 Hz); 131.5; 131.8; HRMS (APCI): *m/z* calcd. for $[C_7H_6Br\text{-}CF_2H]^+$ 168.9647, found 168.9656.

1-(2,2-difluoroethyl)-4-phenoxybenzene (4e)

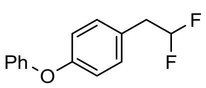

This compound was prepared according to the above general procedure. The product was isolated as colorless oil using pentane:dichloromethane, 5:1 as eluent system (13 mg, 55 %). ^{1}H-NMR (400 MHz, CDCl$_3$) δ 3.14 (td, J_{HF} = 17.3, J = 4.4 Hz, 2H); 5.94 (tt, J_{HF} = 56.6, J = 4.5 Hz, 1H); 6.98-7.05 (m, 4H); 7.11-7.15 (m, 1H); 7.22-7.25 (m, 2H); 7.34-7.38 (m, 2H); ^{19}F-NMR (376 MHz, CDCl$_3$) δ -115.04 (dt, J_{HF} = 56.5, 17.4 Hz); ^{13}C-NMR (100 MHz, CDCl$_3$) δ 40.3 (t, J_{CF} = 22.9 Hz); 116.6 (t, J_{CF} = 242.8 Hz); 118.9; 119.0; 123.4; 127.1 (t, J_{CF} = 5.9 Hz); 129.8; 131.1; 156.8; 157.0; HRMS (APCI): *m/z* calcd. for $[C_{14}H_{12}F_2O+H]^+$ 235.0929, found 235.0935.

Methyl 4-(2,2-difluoroethyl)benzoate (4f)

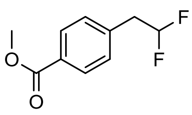

This compound was prepared according to the above general procedure. The product was isolated as colorless oil using pentane: dichloromethane, 2:1 as eluent system (13 mg, 65%). ^{1}H-NMR (400 MHz, CDCl$_3$) δ 3.32 (td, J_{HF} = 17.1, J = 4.5 Hz, 2H); 3.94 (s, 3H); 5.97 (tt, J_{HF} = 56.3, J = 4.5 Hz, 1H); 7.34-7.36 (m, 2H); 8.02-8.05 (m, 2H); ^{19}F-NMR (376 MHz, CDCl$_3$) δ -114.86 (dt, J_{HF} = 56.3, 17.2 Hz); ^{13}C-NMR (100 MHz, CDCl$_3$) δ 40.8 (t, J_{CF} = 22.3 Hz); 52.2; 116.0 (t, J_{CF} = 241.3 Hz); 129.5, 129.9; 129.94; 137.5 (t, J_{CF} = 5.6 Hz); 166.8; HRMS (ESI): *m/z* calcd. for $[C_{10}H_{10}F_2O_2+Na]^+$ 223.0541, found 223.0539.

3-(2,2-difluoroethyl)-1,1'-biphenyl (4h)

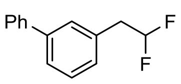

This compound was prepared according to the above general procedure. The product was isolated as colorless oil using pentane: dichloromethane, 5:1 as eluent system (13 mg, 60%).^{1}H-NMR (400 MHz, CDCl$_3$) δ 3.24 (td, J_{HF} = 17.3, J = 4.4 Hz,

2H); 6.00 (tt, J_{HF} = 56.5, J = 4.6 Hz, 1H); 7.24-7.28 (m, 1H); 7.35-7.41 (m, 1H); 7.42-7.51 (m, 4H); 7.53-7.57 (m, 1H); 7.58-7.64 (m, 2H); ^{19}F-NMR (376 MHz, $CDCl_3$) δ -114.68 (dt, J_{HF} = 56.5, 17.4 Hz); ^{13}C-NMR (100 MHz, $CDCl_3$) δ 41.0 (t, J_{CF} = 22.0 Hz); 116.6 (t, J_{CF} = 241.7 Hz); 126.4; 127.2; 127.5; 128.6; 128.7; 128.8; 129.1; 132.9 (t, J_{CF} = 5.8 Hz); 140.8; 141.8; HRMS (APCI): *m/z* calcd. for $[C_{14}H_{12}F_2+H]^+$ 219.0980, found 219.0981.

1-Bromo-3-(2,2-difluoroethyl)benzene (4i)

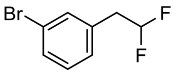

This compound was prepared according to the above general procedure. The product was isolated as colorless oil using pentane as eluent system (12 mg, 54%). ^{1}H-NMR (400 MHz, $CDCl_3$) δ 3.14 (td, J_{HF} = 17.1, J = 4.4 Hz, 2H); 5.95 (tt, J_{HF} = 56.2, J = 4.8 Hz, 1H); 7.20-7.26 (m, 2H); 7.44-7.47 (m, 2H); ^{19}F-NMR (376 MHz, $CDCl_3$) δ -115.00 (dt, J_{HF} = 56.8, 17.0 Hz); ^{13}C-NMR (100 MHz, $CDCl_3$) δ 40.7 (t, J_{CF} = 21.7 Hz); 116.1 (t, J_{CF} = 241.7 Hz); 122.8; 128.5; 130.3; 130.7; 132.9; 134.6 (t, J_{CF} = 5.7 Hz); HRMS (APCI): *m/z* calcd. for $[C_7H_6Br\text{-}CF_2H]^+$ 168.9647, found 168.9651.

4-(2,2-difluoropropyl)-1,1'-biphenyl (4j)

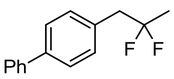

This compound was prepared according to the above general procedure. The product was isolated as white solid using pentane:dichloromethane, 5:1 as eluent system (15 mg, 65 %). ^{1}H-NMR (400 MHz, $CDCl_3$) δ 1.58 (t, J_{HF} = 18.3 Hz, 3H); 3.19 (t, J_{HF} = 15.5 Hz, 2H); 7.38-7.32 (m, 3H); 7.49-7.40 (m, 2H); 7.62-7.54 (m, 4H); ^{19}F-NMR (376 MHz, $CDCl_3$) δ -88.84 - -89.12 (m). ^{13}C-NMR (100 MHz, $CDCl_3$) δ 22.9 (t, J_{CF} = 27.5 Hz); 44.1 (t, J_{CF} = 26.3 Hz); 123.4 (t, J_{CF} = 239.6 Hz); 127.1; 127.2; 127.3; 128.8; 130.6; 132.7 (t, J_{CF} = 5.0 Hz); 140.3; 140.7; HRMS (APCI): *m/z* calcd. for $[C_{15}H_{14}F_2+H]^+$ 233.1136, found 233.1144; Mp: 72-73 ^{o}C.

2-(2,2-difluoropropyl)naphthalene (4k)

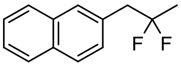

This compound was prepared according to the above general procedure. The product was isolated as colorless oil using pentane:dichloromethane, 5:1 as eluent system (12 mg, 59 %). ^{1}H-NMR (400 MHz, $CDCl_3$) δ 1.57 (t, J_{HF} = 18.4 Hz, 3H); 3.31 (t, J_{HF} = 15.4 Hz, 2H); 7.38-7.43 (m, 1H); 7.53-7.44 (m, 2H); 7.73 (s, 1H); 7.87-7.79 (m, 3H); ^{19}F-NMR (376 MHz, $CDCl_3$) δ -88.42 - -88.70 (m); ^{13}C-NMR (100 MHz, $CDCl_3$) δ 23.1 (t, J_{CF} = 27.4 Hz); 44.8 (t, J_{CF} = 26.4 Hz); 123.8 (t, J_{CF} = 239.7 Hz); 126.1; 126.4; 127.8; 127.9; 128.3; 128.4; 129.3; 131.5 (t, J_{CF} = 5.1 Hz); 132.8; 133.5; HRMS (APCI): *m/z* calcd. for $[C_{13}H_{12}F_2+H]^+$ 207.0980, found 207.0987.

1-(2,2-difluoropropyl)-4-phenoxybenzene (4l)

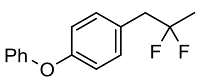

This compound was prepared according to the above general procedure. The product was isolated as colorless oil using pentane: dichloromethane, 5:1 as eluent system (13 mg, 52%).^{1}H-NMR (400 MHz, $CDCl_3$) δ 1.58 (t, J_{HF} = 18.3, 3H); 3.14 (t, J_{HF} = 15.5, 2H); 6.97-7.05 (m, 4H); 7.11-7.15 (m, 1H); 7.23-7.25 (m, 2H); 7.34-7.39 (m, 2H); ^{19}F-NMR (376 MHz, $CDCl_3$) δ -89.27 - -89.50 (m); ^{13}C-NMR (100 MHz, $CDCl_3$) δ 23.0 (t, J_{CF} = 27.5 Hz); 43.8 (t, J_{CF} = 26.4 Hz); 118.8; 119.2; 123.5; 123.6; 128.6 (t, J_{CF} = 5.1 Hz); 129.9; 131.7; 156.8; 157.2; HRMS (APCI): *m/z* calcd. for $[C_{15}H_{14}F_2O]^+$ 248.1007, found 248.1013.

4-(1-fluoro-2-iodoethyl)-1,1'-biphenyl (5)

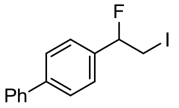

This compound was prepared according to the above general procedure, however $PdCl_2(MeCN)_2$ (5 mg, 0.02 mmol, 20 mol %) was used instead of $AgBF_4$. The product was isolated as pink oil using pentane: dichloromethane, 5:1 as eluent system (14 mg, 43%). N.B. This compound was found to be unstable and decomposed within couple of hours. ^{1}H-NMR (400 MHz, $CDCl_3$) δ 3.54 (m, 2H); 5.62 (ddd, J_{HF} = 46.1, *J* = 7.4, 5.5 Hz, 1H); 7.37-7.39 (m, 1H); 7.40-7.47 (m, 4H); 7.60-7.64 (m, 4H); ^{19}F-NMR (376 MHz, $CDCl_3$) δ -165.85 (ddd, J_{HF} = 46.8, 24.0, 16.0 Hz); ^{13}C-NMR (100 MHz, $CDCl_3$) δ 7.3 (d, J_{CF} = 28.4 Hz); 93.2 (d, J_{CF} = 177.3 Hz); 126.3 (d, J_{CF} = 6.3 Hz); 127.3; 127.6; 127.8; 129.0; 137.0 (d, J_{CF} = 22.2 Hz); 140.5; 142.3 (d, J_{CF} = 1.6 Hz); HRMS (APCI): *m/z* calcd. for $[C_{14}H_{12}\text{-}FI]^+$ 180.0934, found 180.0940. Comparision of the above ^{1}H/^{19}F NMR data for **5** with NMR data published[3] for the very close analog, (1-fluoro-2-iodoethyl)benzene, clearly indicates the presence of iodine in **5**. Thus the literature shift value[3] for the $C\underline{H}_2I$ protons in (1-fluoro-2-iodoethyl)benzene is 3.55-3.41 ppm and the ^{19}F shift is -166.4 ppm). The chloro analog, (2-chloro-1-fluoroethyl)benzene has a significantly different shift value for the $C\underline{H}_2Cl$ protons (3.86-3.96 ppm) and for the ^{19}F shift (-178.4 ppm).

Competitive difluorination

The competitive difluorination reaction was performed under the reaction conditions given in the above general procedure. Thus, iodane reagent **1** (28.0 mg, 0.1 mmol), styrenes **2a** (18 mg, 0.1 mmol), **2a-**d_2 (18 mg, 0.1 mmol) and $AgBF_4$ **3** (19.4 mg, 0.1 mmol) were mixed in $CDCl_3$ (0.5 ml). This reaction mixture was stirred at 40°C for 18 h. According to NMR products **4a** and **4a-**$\mathbf{d_2}$ were formed (1.3:1 ratio), indicating a weak isotope effect of 1.3.

References

(1) a) G. C. Geary, E. G. Hope, K. Singh, A. M. Stuart, *Chem. Commun.* **2013**, *49*, 9263.
b) V. Matoušek, E. Pietrasiak, R. Schwenk, A. Togni, *J. Org. Chem.* **2013**, *78*, 6763

(2) D. H. T. Phan, K. G. M. Kou, V. M. Dong, *J. Am. Chem. Soc.* **2010**, *132*, 16354.

(3) H. Yoshino, K. Matsumoto, R. Hagiwara, Y. Ito, K. Oshima, S. Matsubara, *J. Fluorine Chem*. **2006**, *127*, 29.

NMR spectra of products 2a-d$_2$ and 4a-l

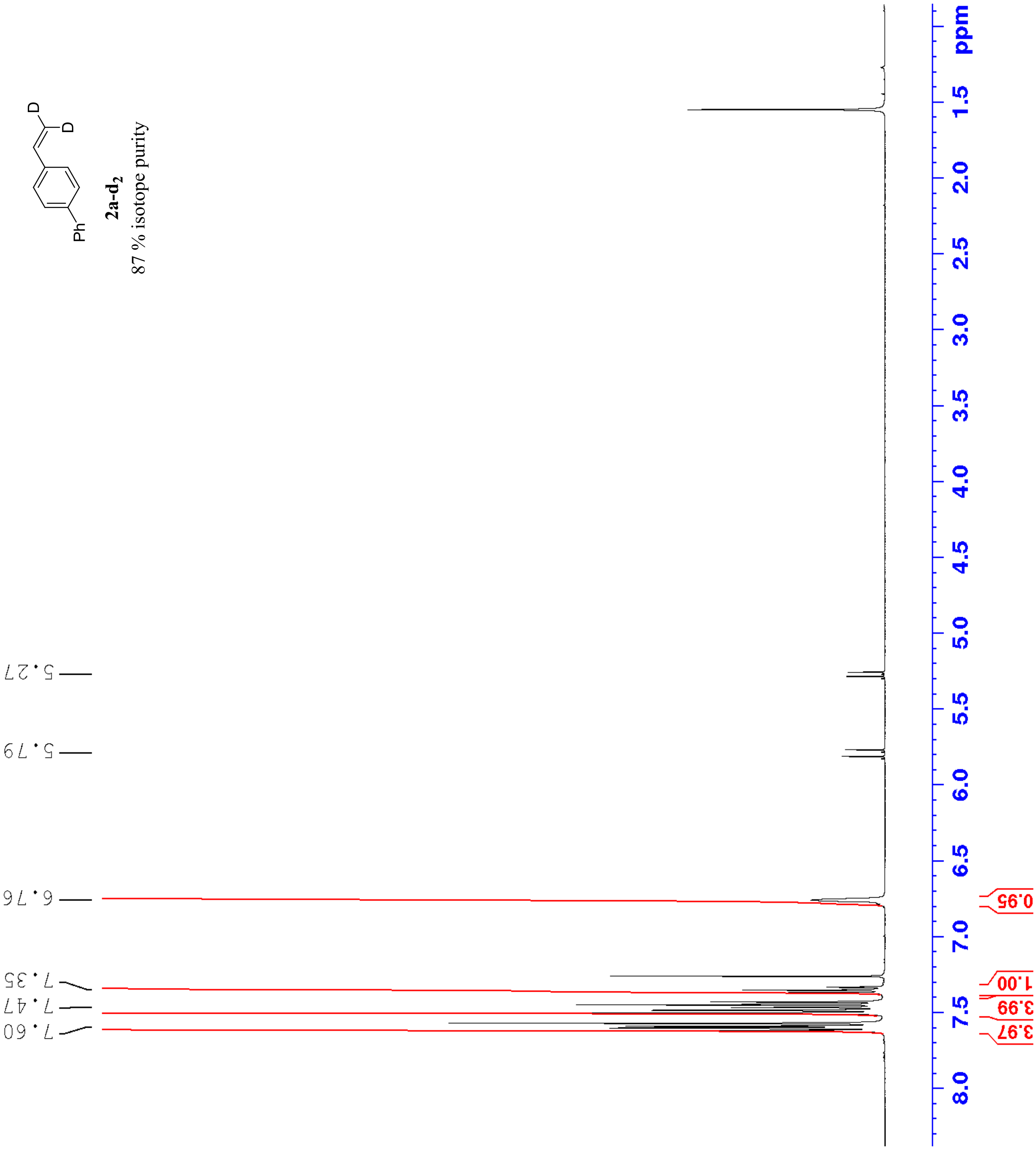

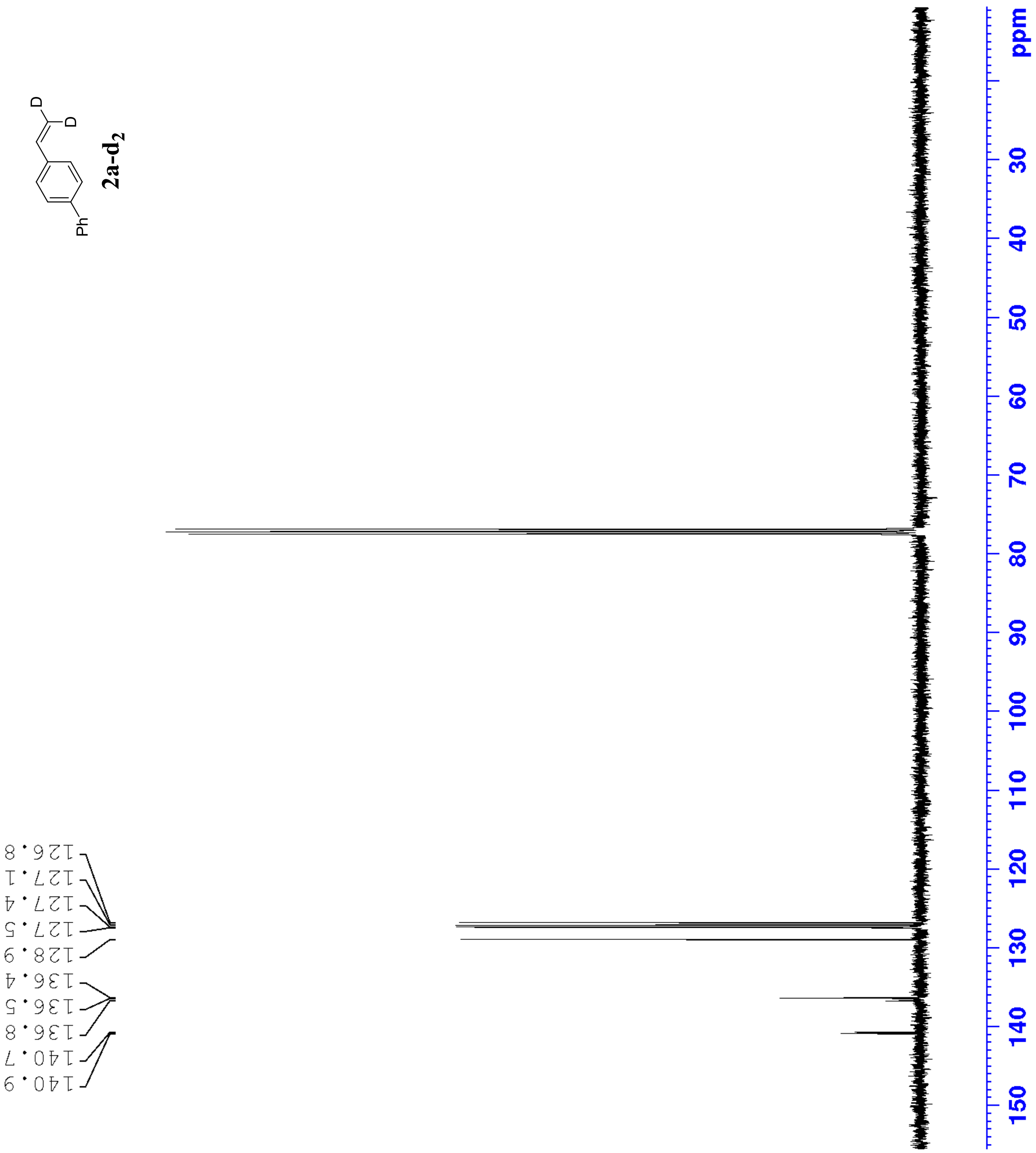
2a-d2
Ph
D
D
126.8
127.1
127.4
127.5
128.9
136.4
136.5
136.8
140.7
140.9
ppm
30
40
50
60
70
80
90
100
110
120
130
140
150

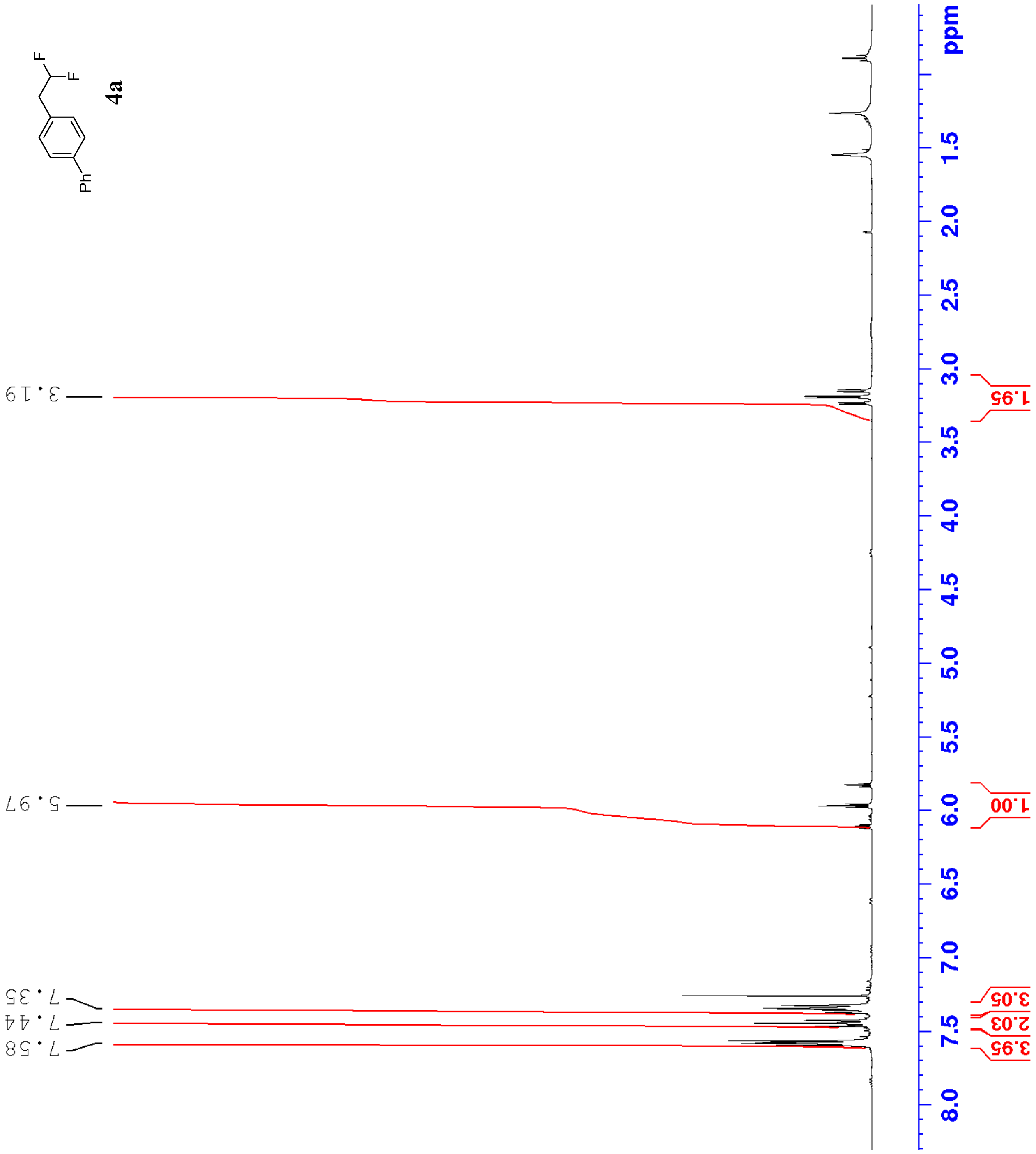

Ph
F
F
4a
3.19
5.97
7.35
7.44
7.58
1.95
1.00
3.05
2.03
3.95
ppm
1.5
2.0
2.5
3.0
3.5
4.0
4.5
5.0
5.5
6.0
6.5
7.0
7.5
8.0

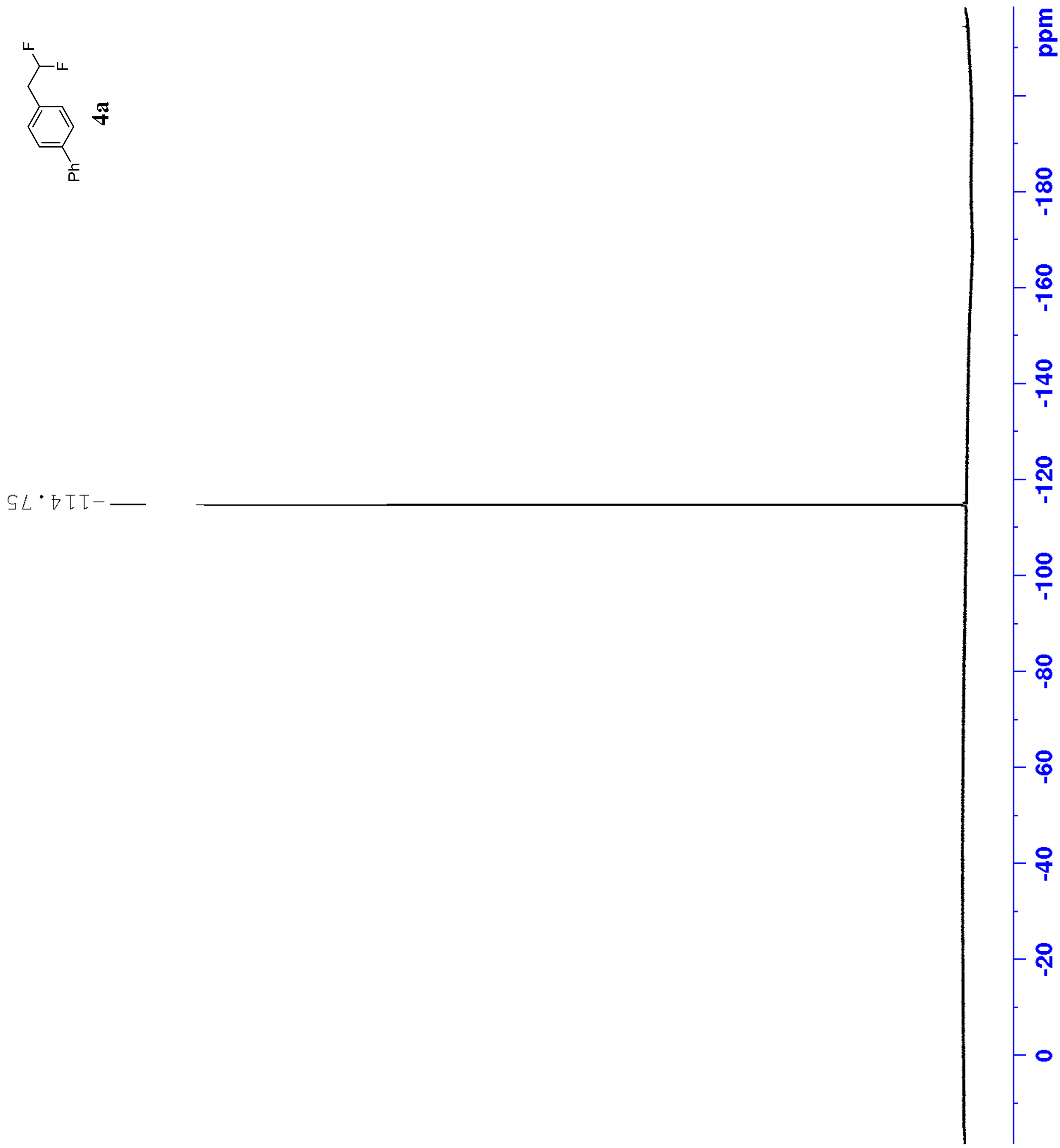
Ph
F
F
4a
-114.75
ppm
-180
-160
-140
-120
-100
-80
-60
-40
-20
0

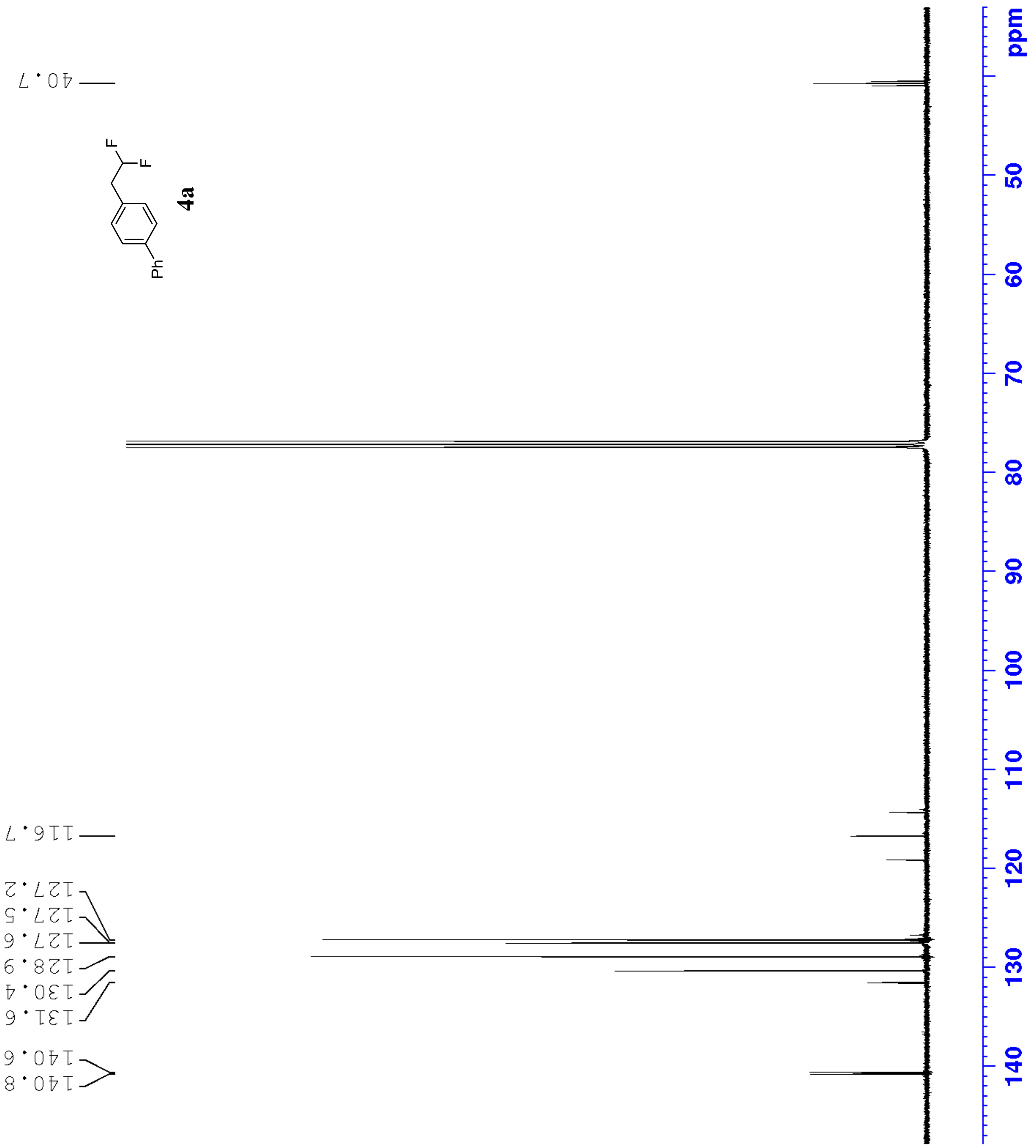
40.7
116.7
127.2
127.5
127.6
128.9
130.4
131.6
140.6
140.8
F
F
Ph
ppm
50
60
70
80
90
100
110
120
130
140

4a

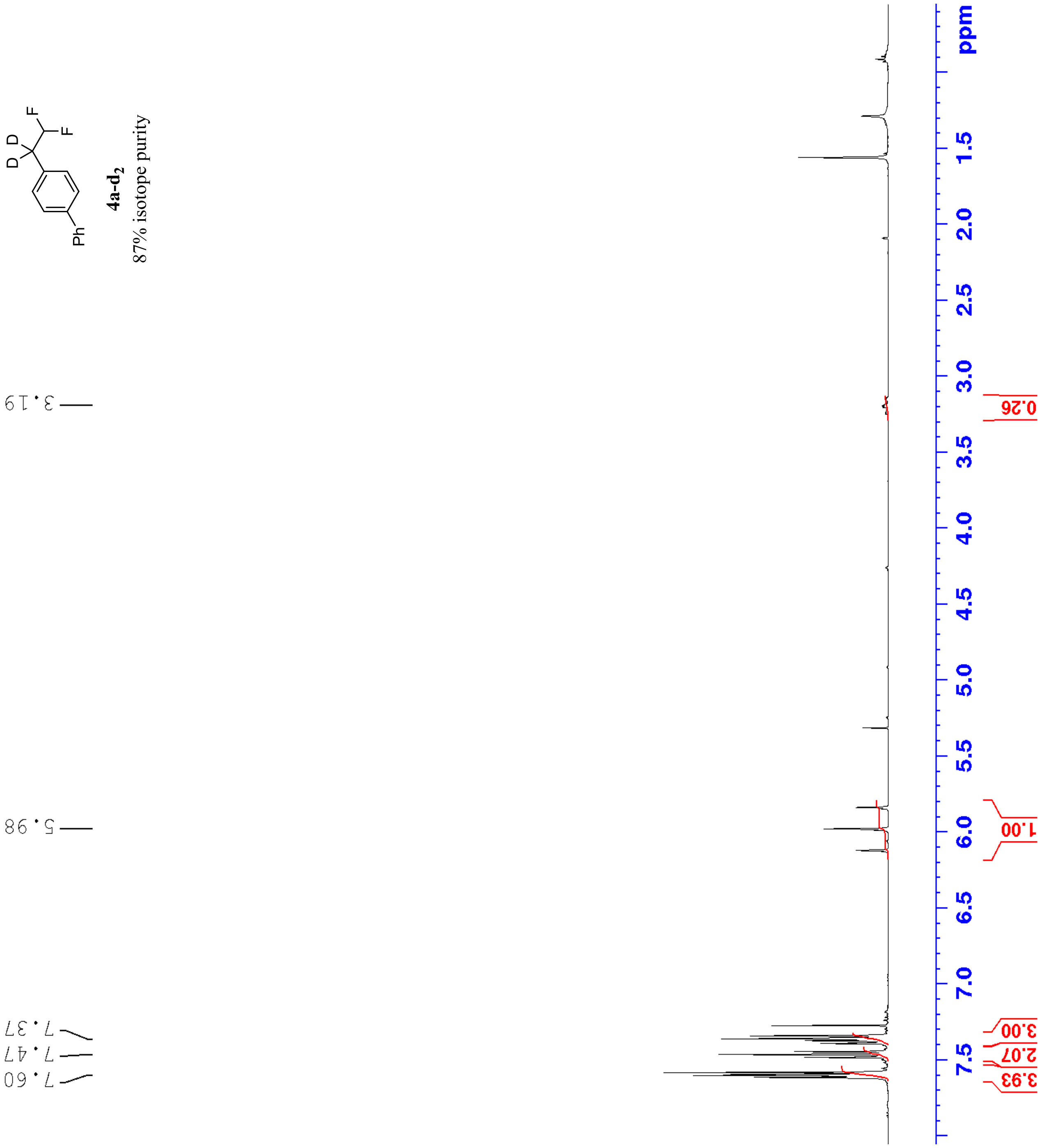

Ph
D D
F
F
4a-d2
87% isotope purity
3.19
5.98
7.37
7.47
7.60
0.26
1.00
3.00
2.07
3.93
ppm
1.5
2.0
2.5
3.0
3.5
4.0
4.5
5.0
5.5
6.0
6.5
7.0
7.5

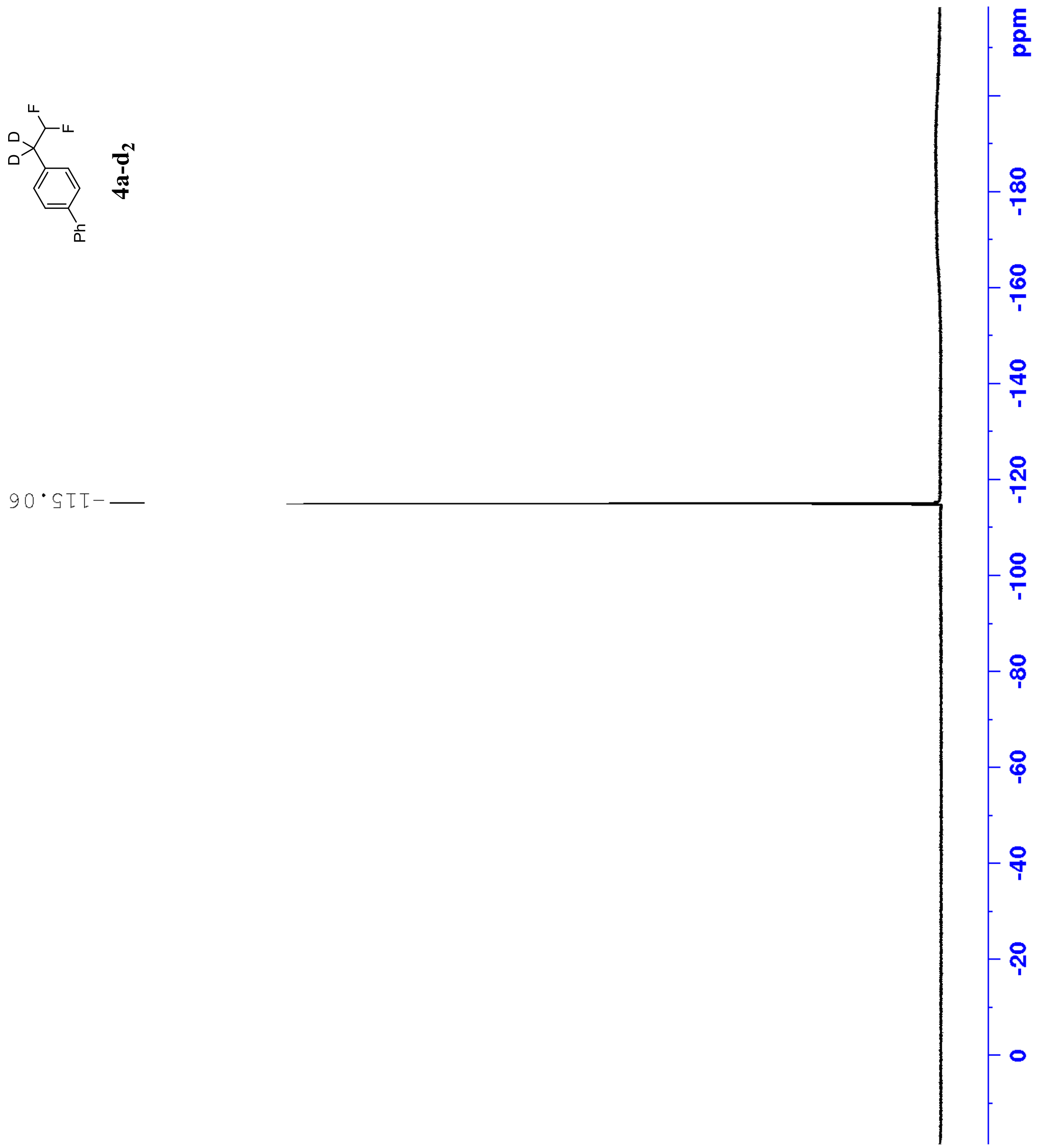
4a-d_2
-115.06
ppm
-180
-160
-140
-120
-100
-80
-60
-40
-20
0

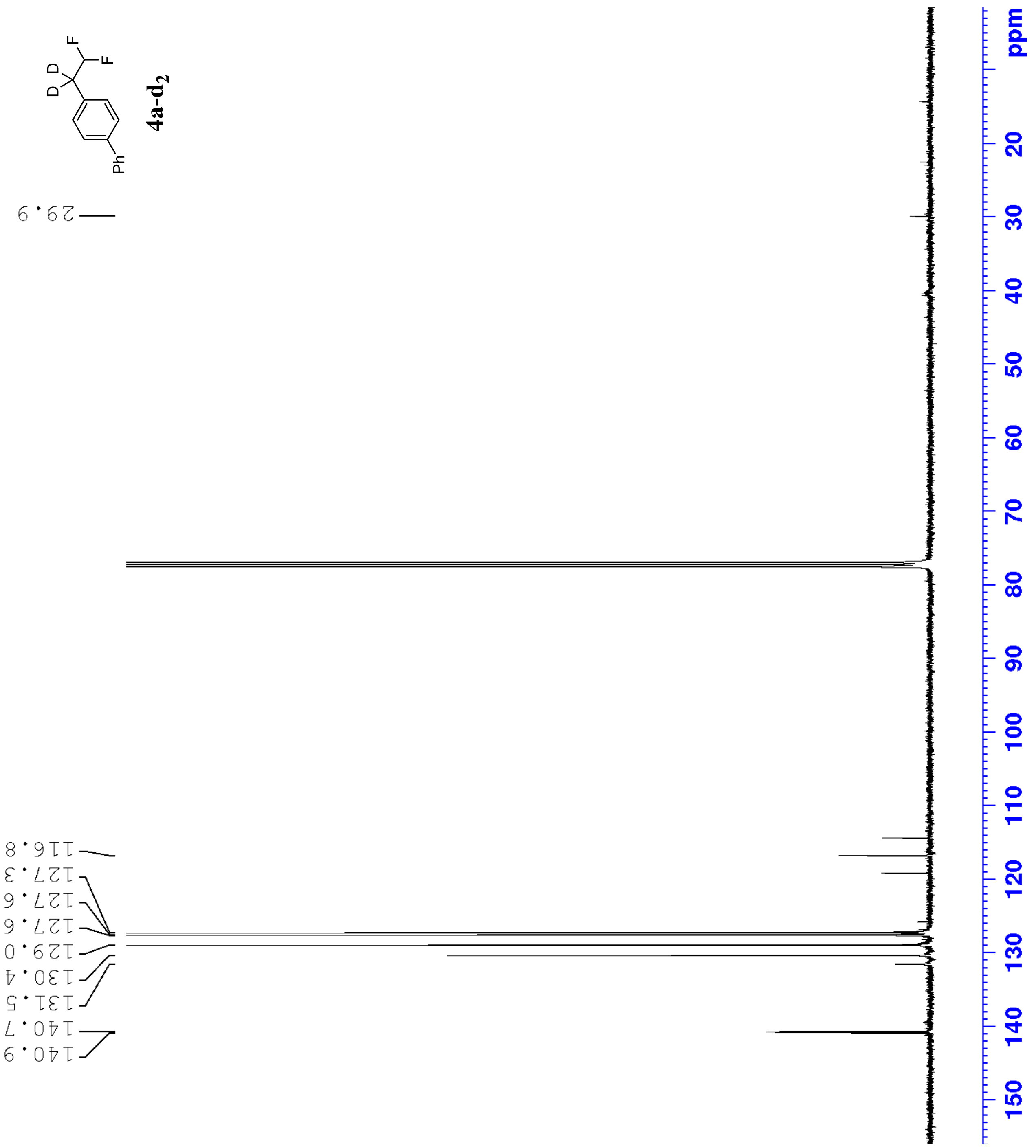

4a-d$_2$
Ph
D
D
F
F
29.9
116.8
127.3
127.6
127.6
129.0
130.4
131.5
140.7
140.9
ppm
20
30
40
50
60
70
80
90
100
110
120
130
140
150

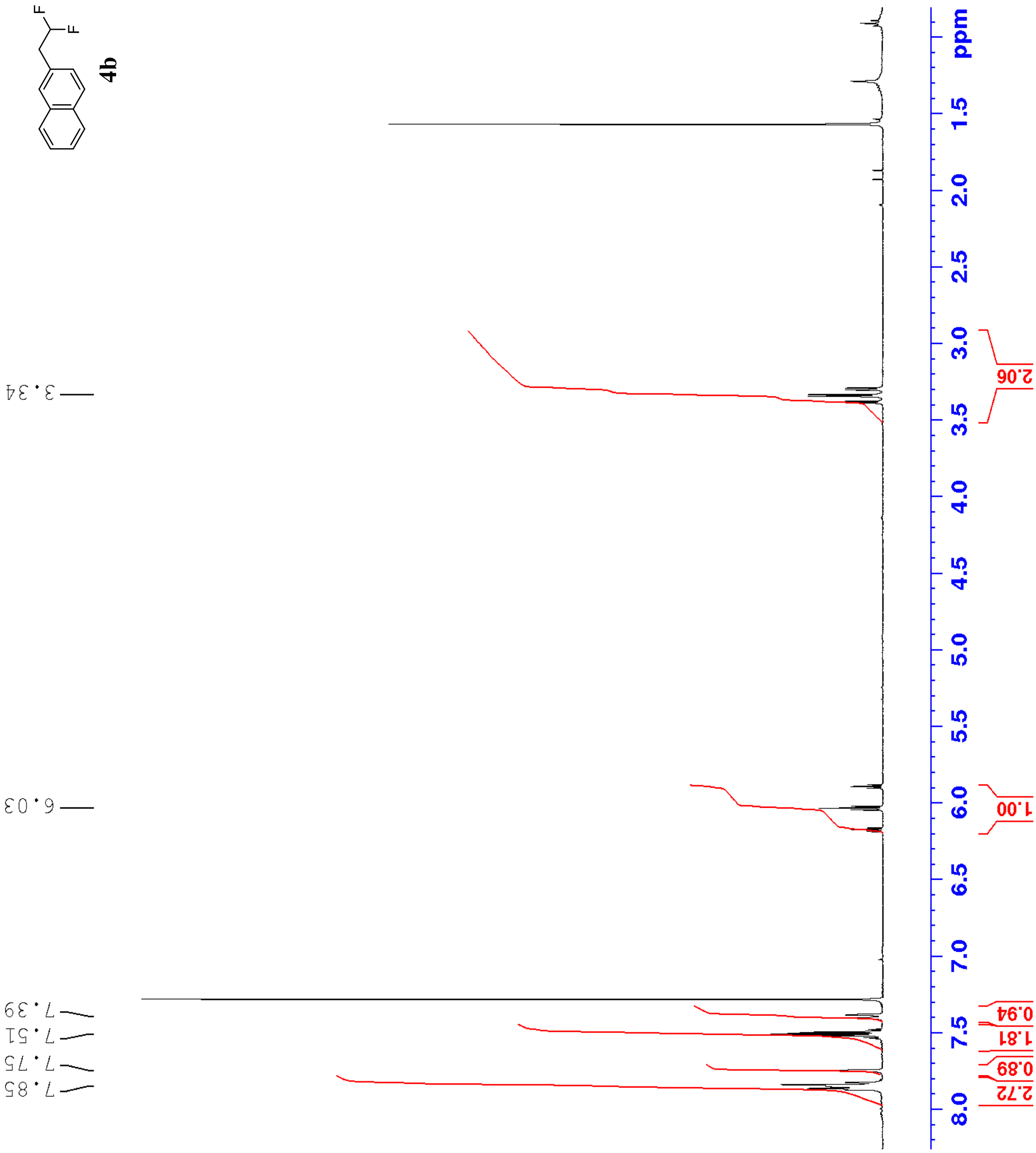
4b
3.34
6.03
7.39
7.51
7.75
7.85
2.06
1.00
0.94
1.81
0.89
2.72
ppm

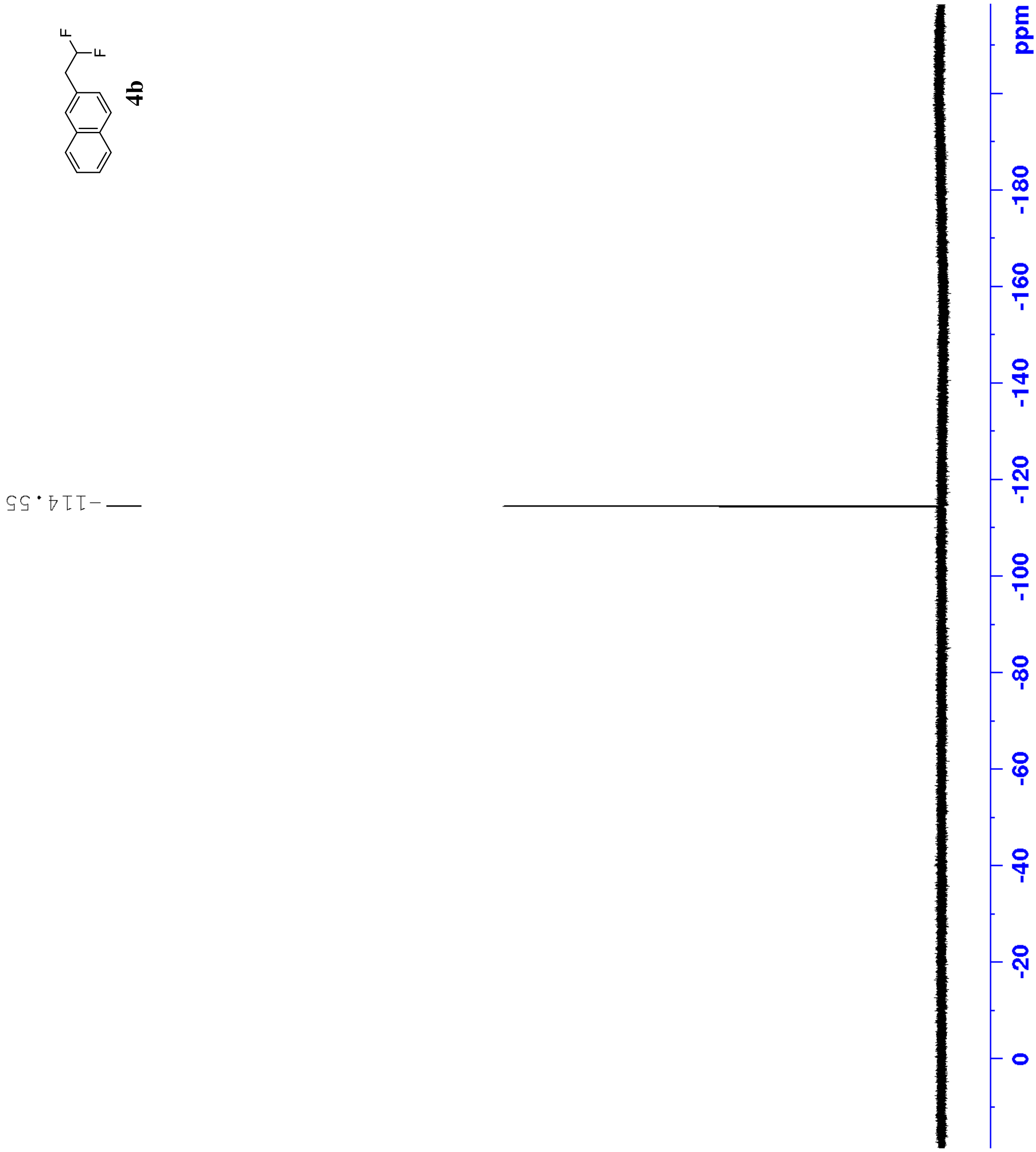
4b
-114.55
ppm
-180
-160
-140
-120
-100
-80
-60
-40
-20
0

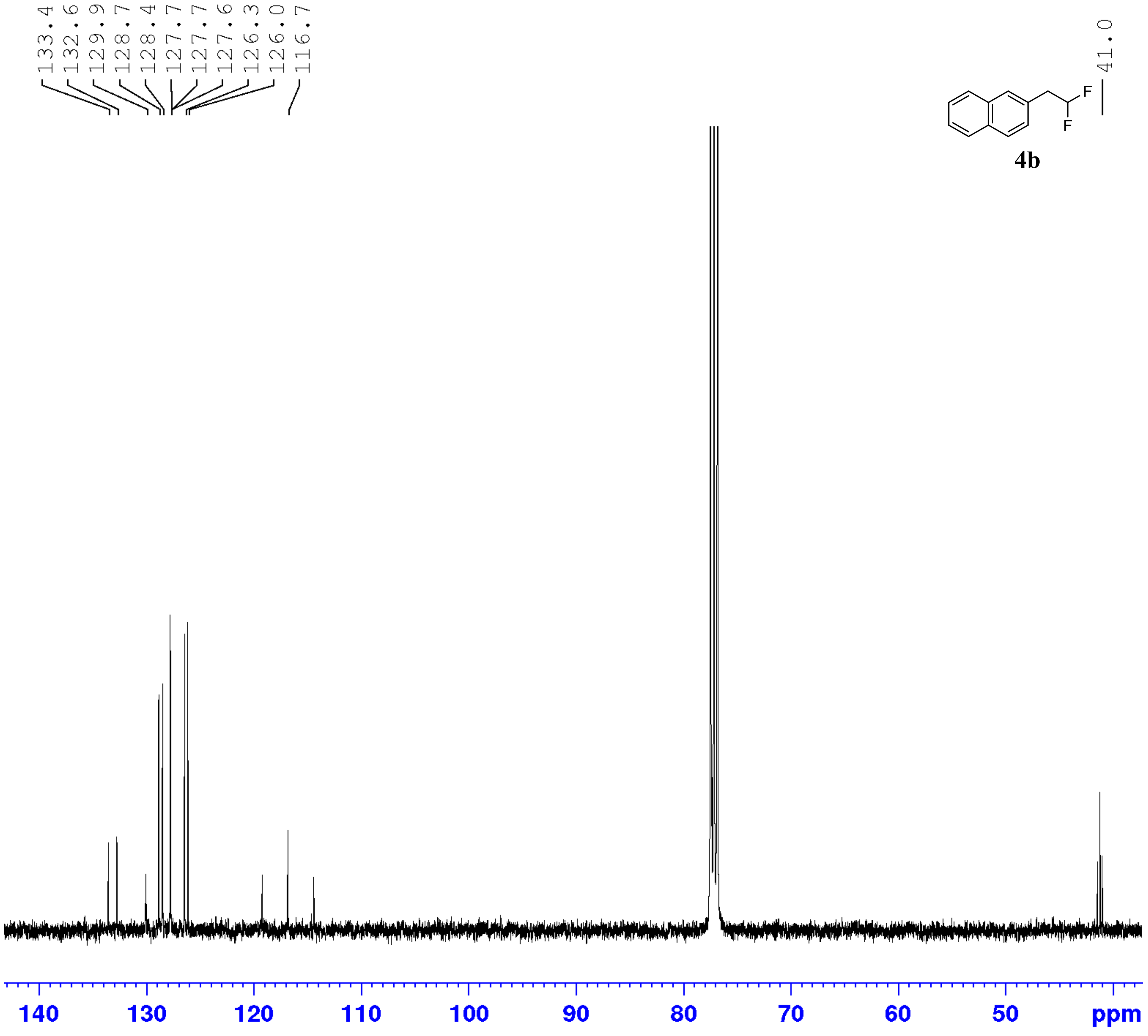
133.4
132.6
129.9
128.7
128.4
127.7
127.7
127.6
126.3
126.0
116.7
41.0
F
F
140
130
120
110
100
90
80
70
60
50
ppm

4b

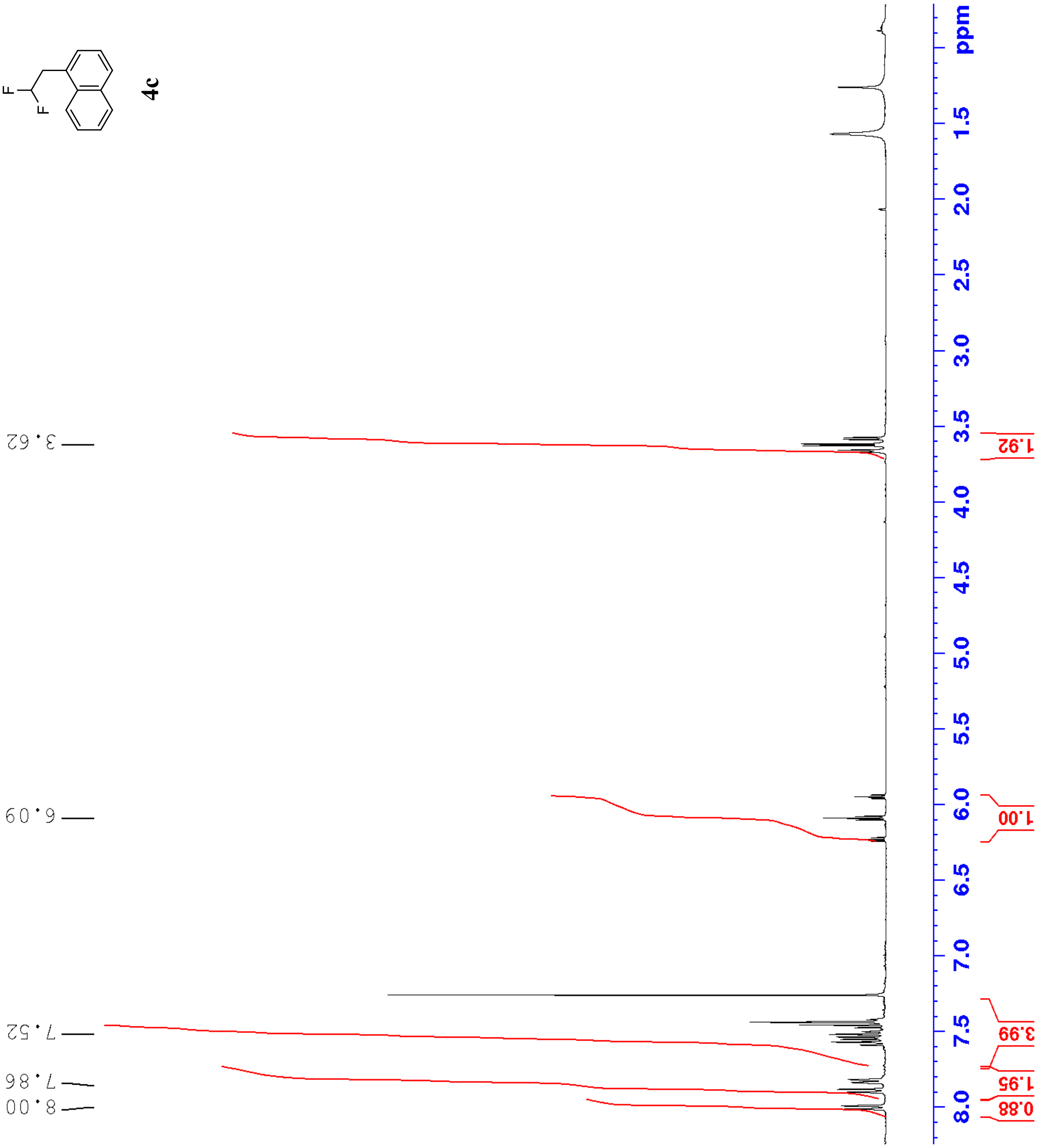

4c
3.62
6.09
7.52
7.86
8.00
ppm
1.5
2.0
2.5
3.0
3.5
4.0
4.5
5.0
5.5
6.0
6.5
7.0
7.5
8.0
1.92
1.00
3.99
1.95
0.88

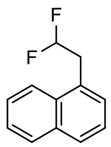

4c

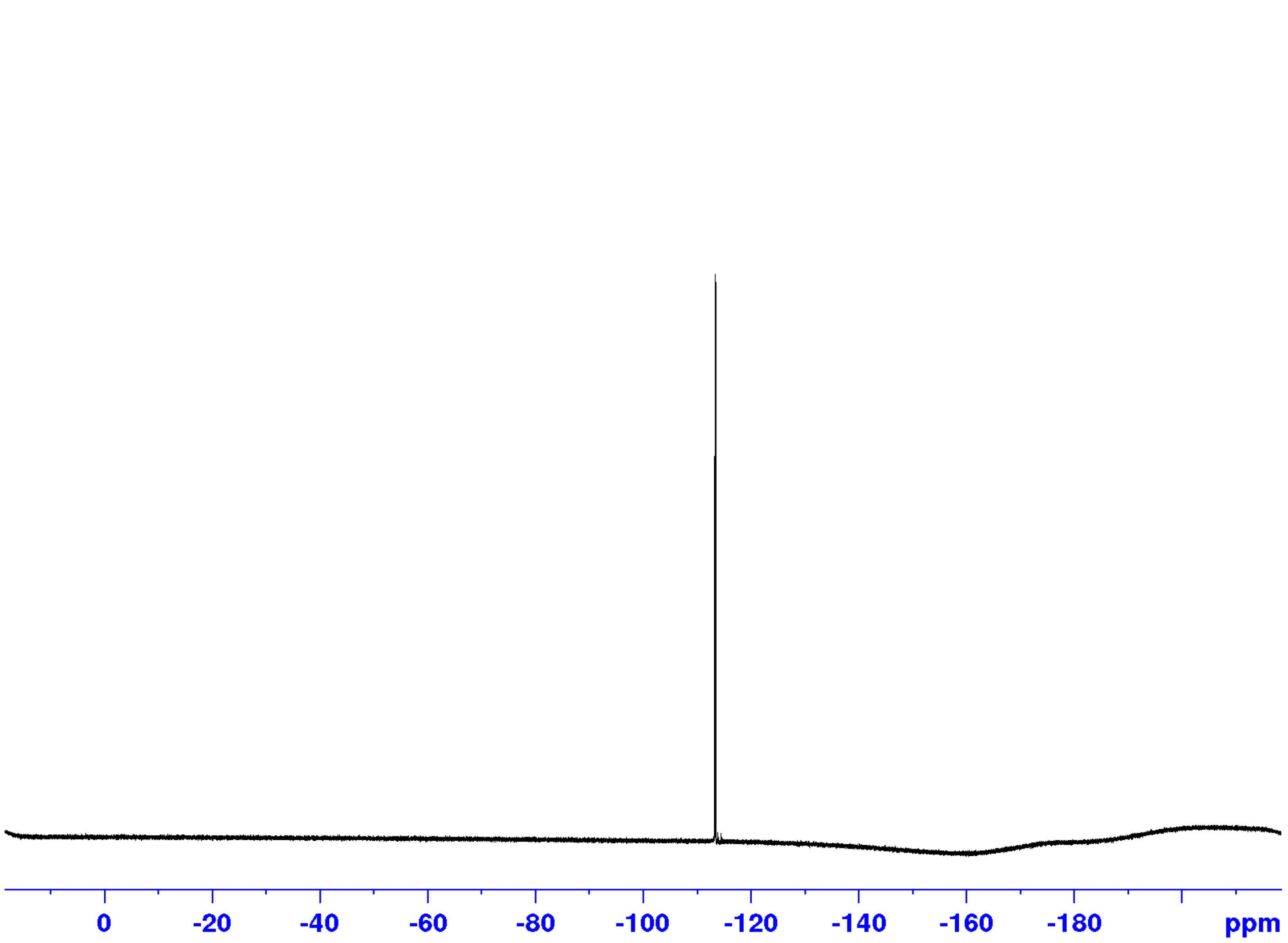

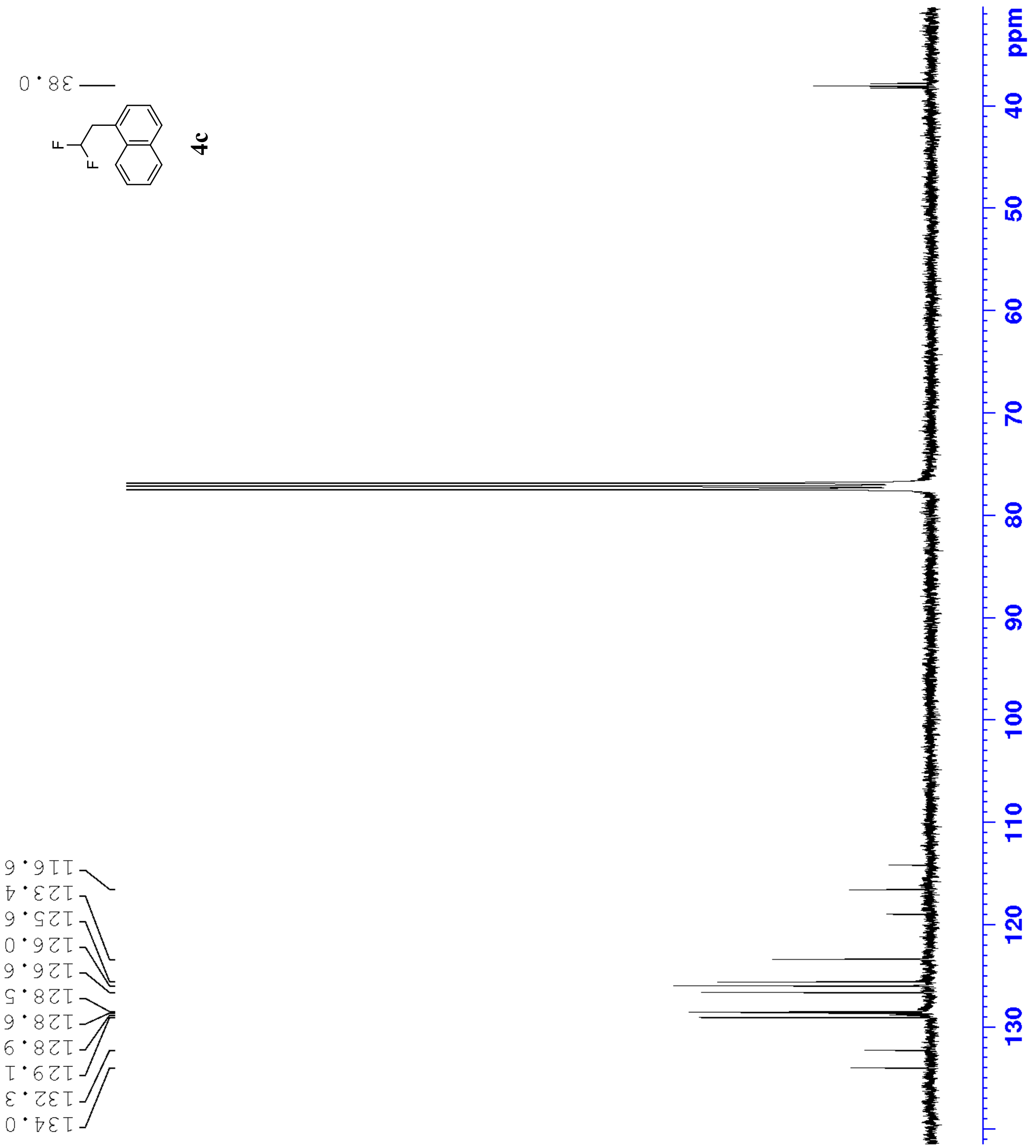
38.0
116.6
123.4
125.6
126.0
126.6
128.5
128.6
128.9
129.1
132.3
134.0
4c
F
F
ppm
40
50
60
70
80
90
100
110
120
130

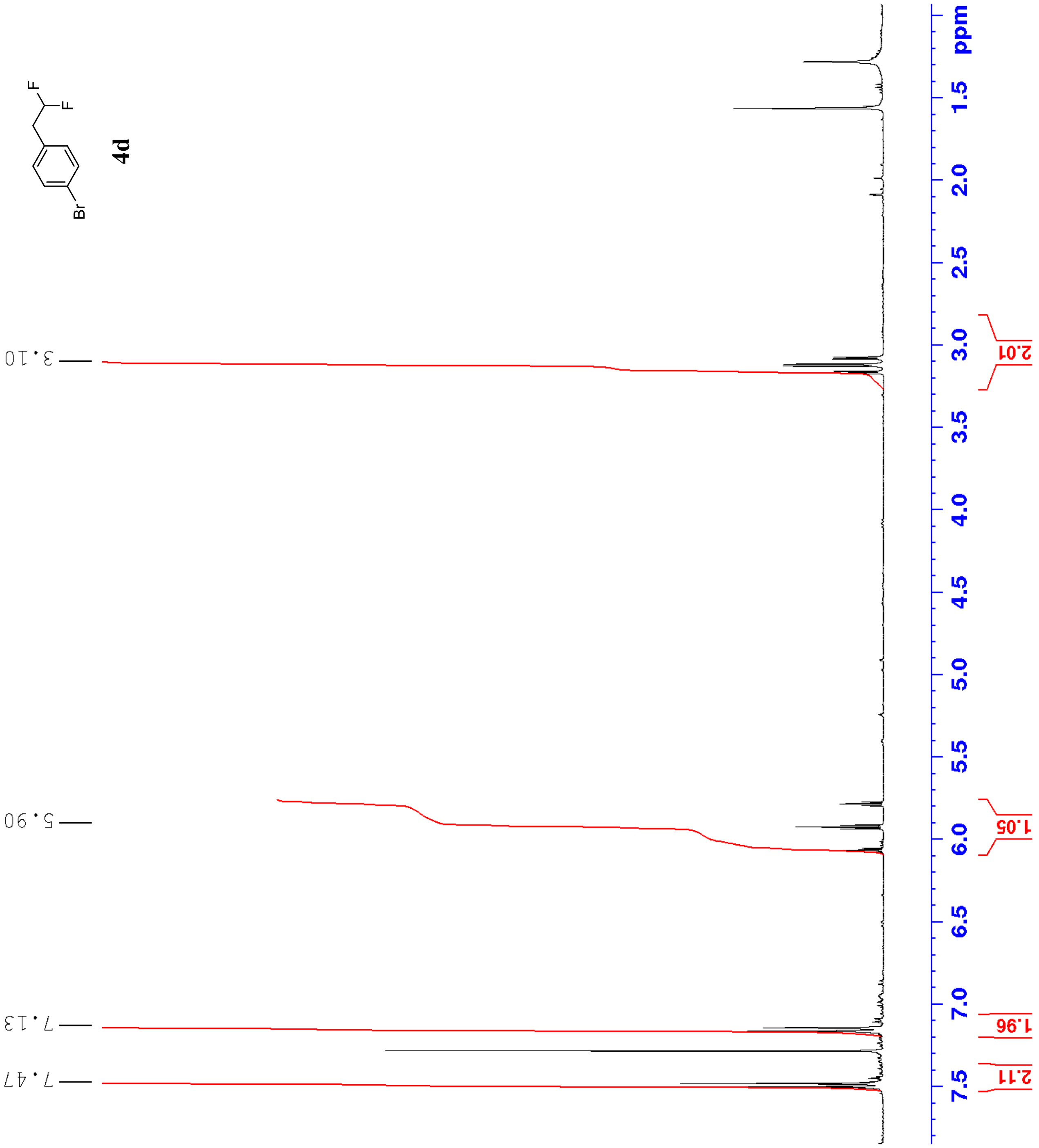
4d
Br
F
F
3.10
5.90
7.13
7.47
2.01
1.05
1.96
2.11
ppm
1.5
2.0
2.5
3.0
3.5
4.0
4.5
5.0
5.5
6.0
6.5
7.0
7.5

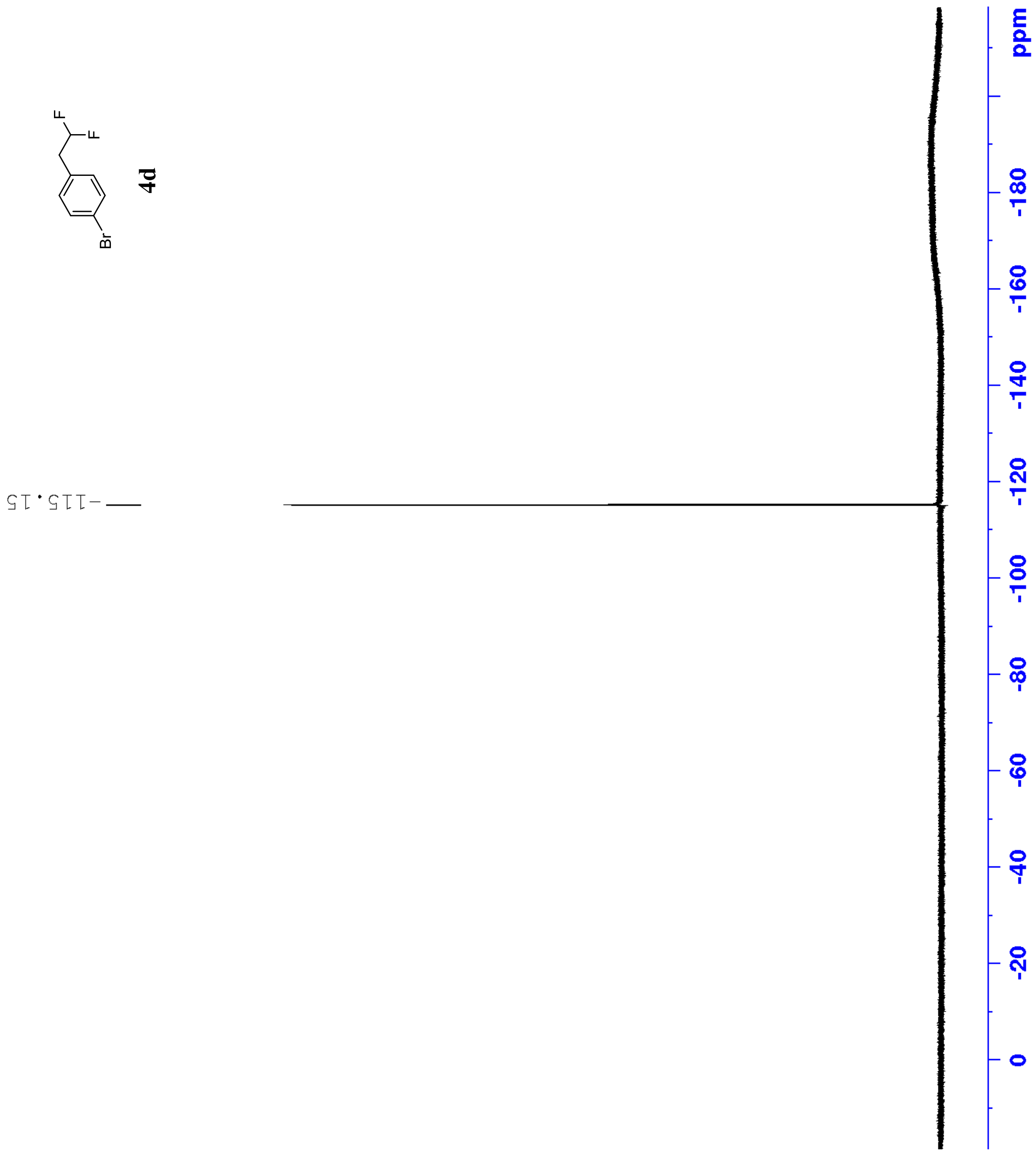
F
F
Br
4d
-115.15
ppm
-180
-160
-140
-120
-100
-80
-60
-40
-20
0

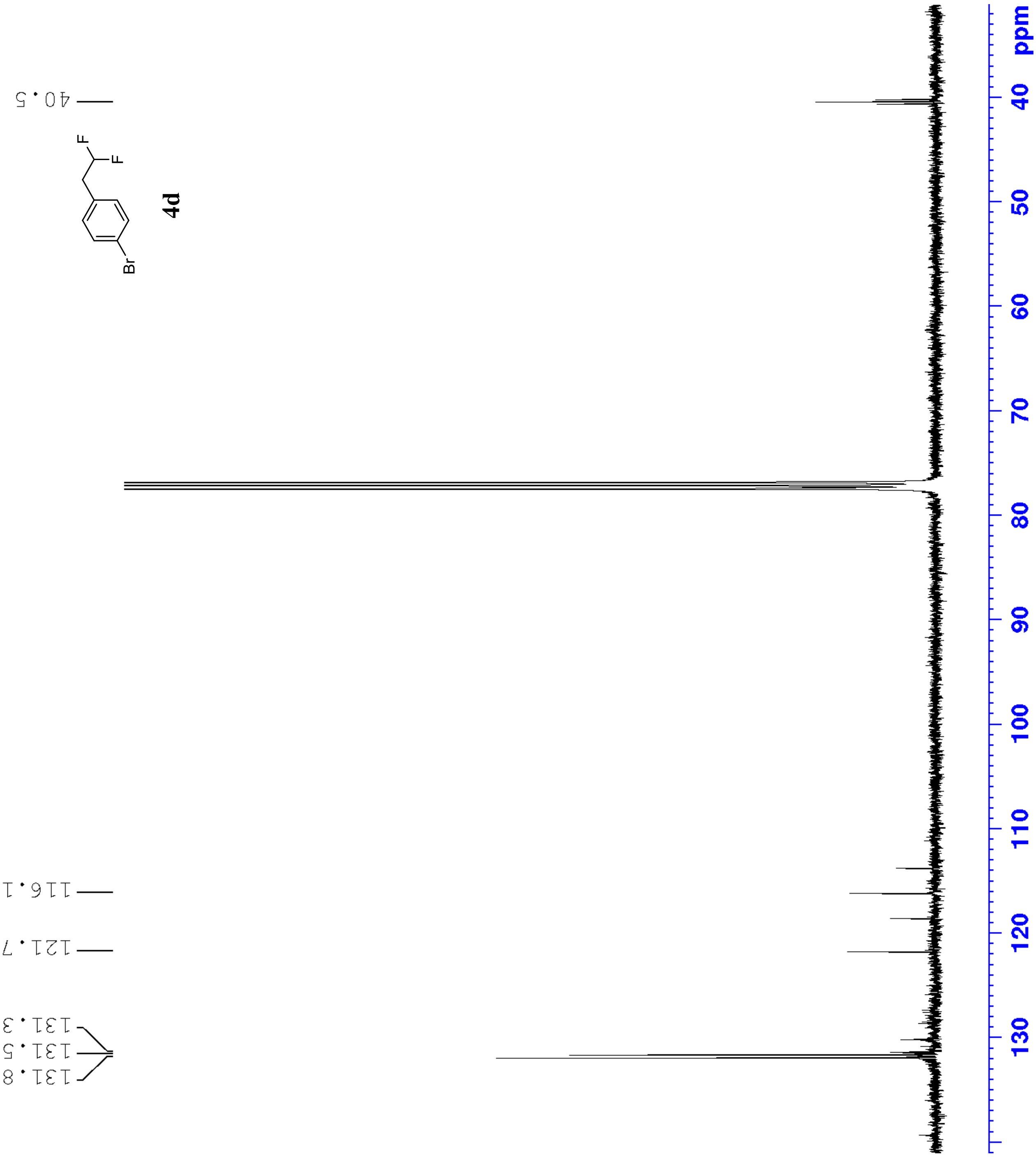
40.5
116.1
121.7
131.3
131.5
131.8
Br
F
F
4d
ppm
40
50
60
70
80
90
100
110
120
130

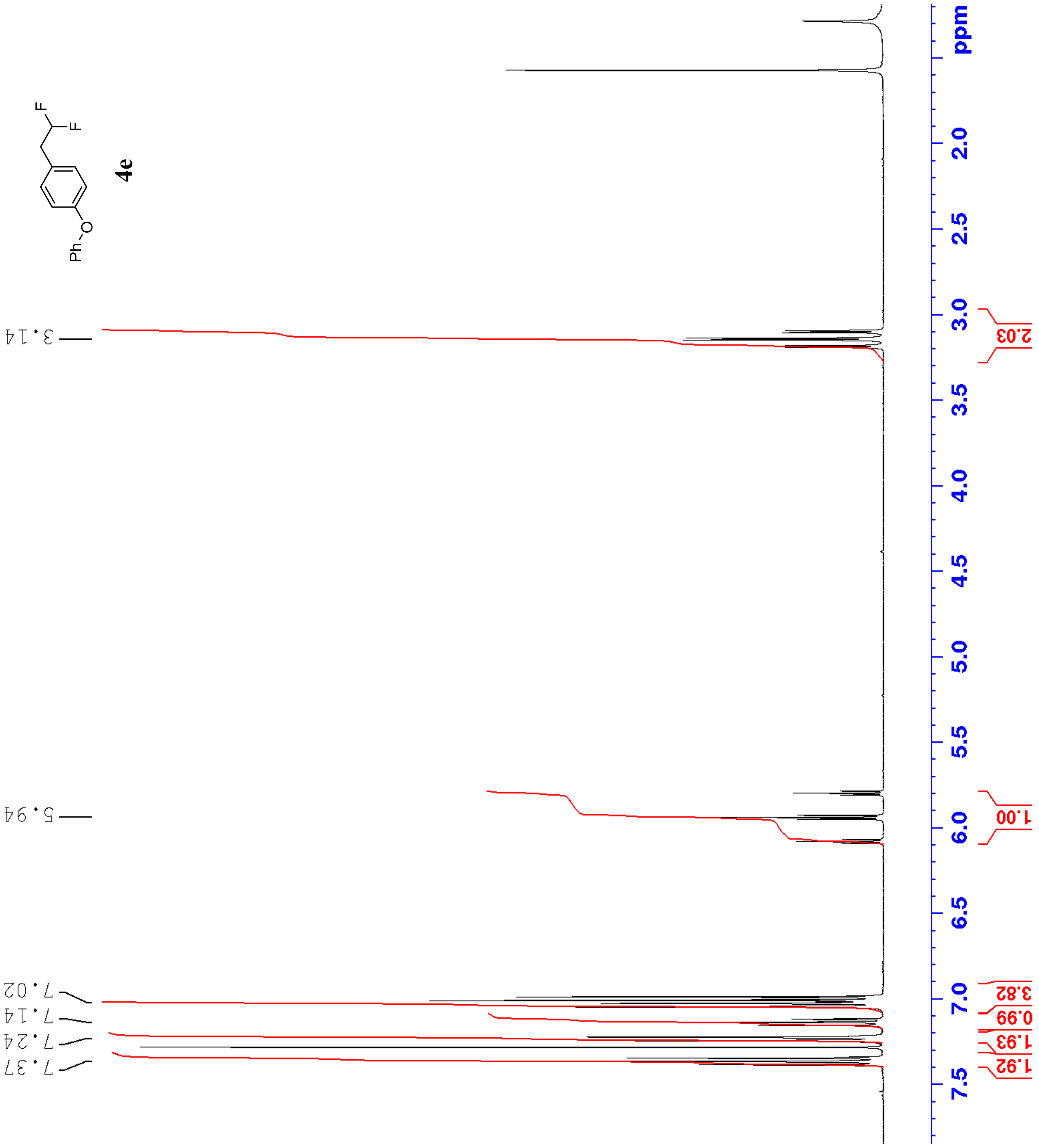

Ph
O
F
F
4e
3.14
5.94
7.02
7.14
7.24
7.37
ppm
2.0
2.5
3.0
3.5
4.0
4.5
5.0
5.5
6.0
6.5
7.0
7.5
2.03
1.00
3.82
0.99
1.93
1.92

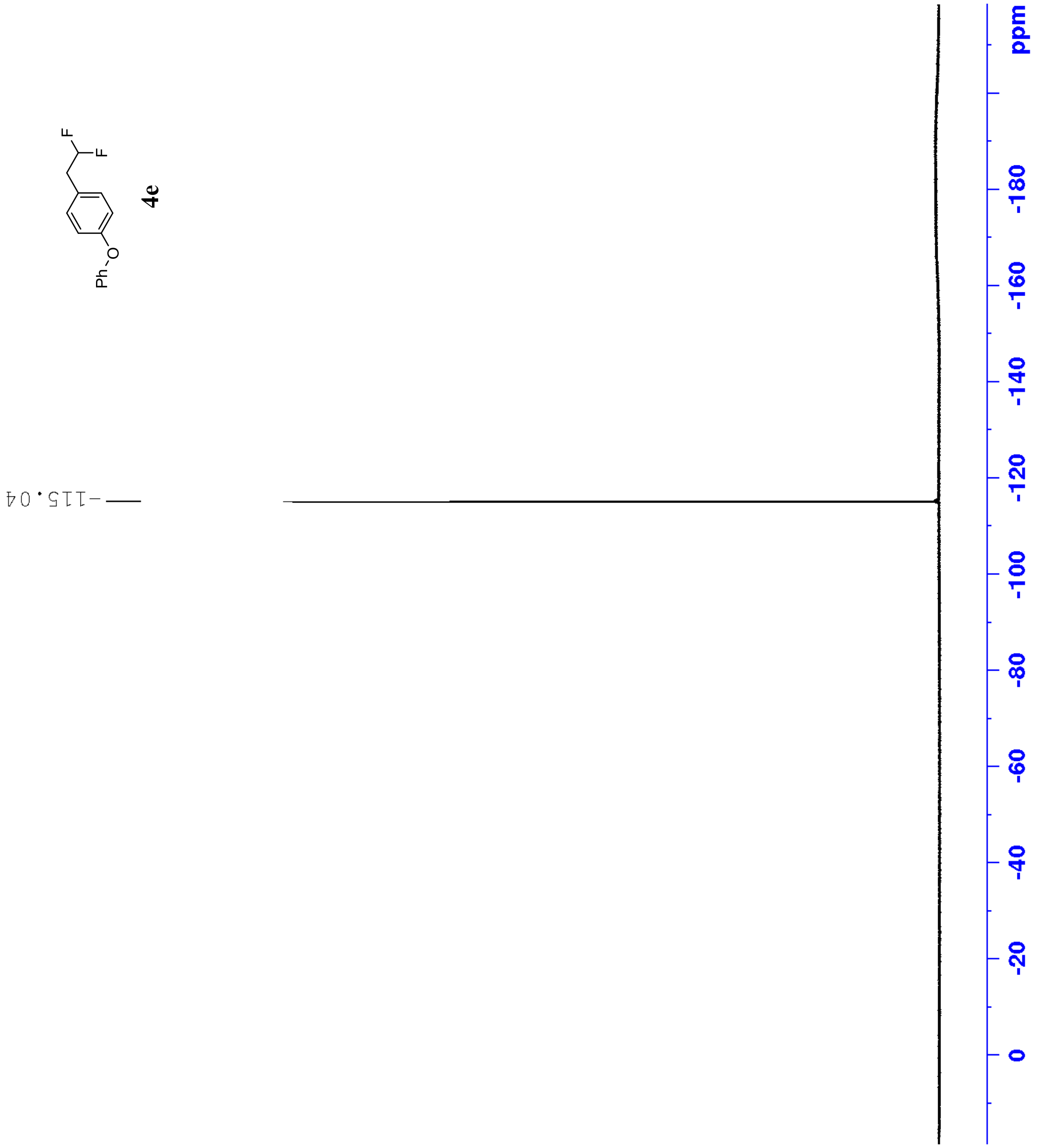

F
F
Ph
O
4e
-115.04
ppm
-180
-160
-140
-120
-100
-80
-60
-40
-20
0

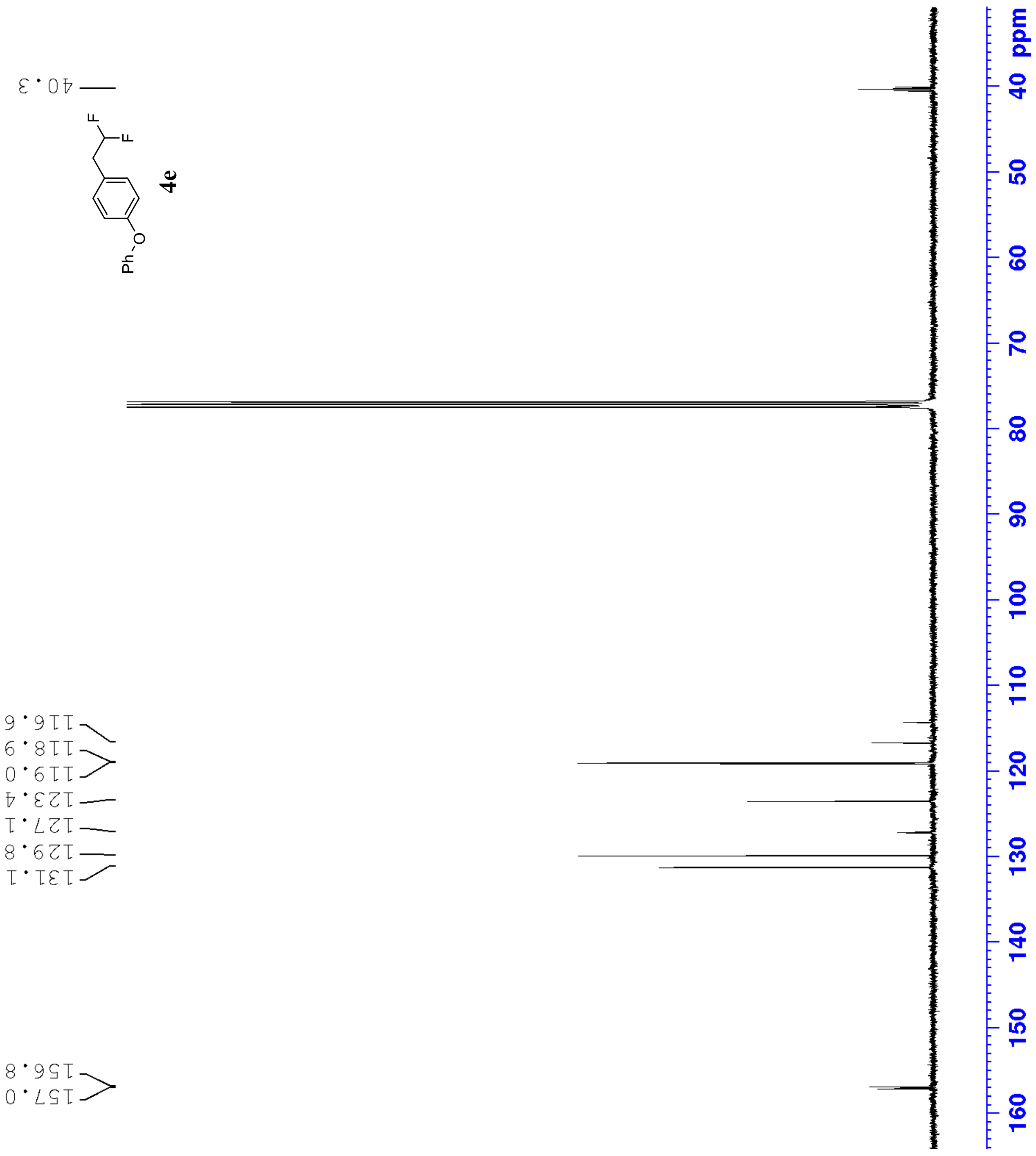

40.3
116.6
118.9
119.0
123.4
127.1
129.8
131.1
156.8
157.0
F
F
Ph
O
4e
ppm
40
50
60
70
80
90
100
110
120
130
140
150
160

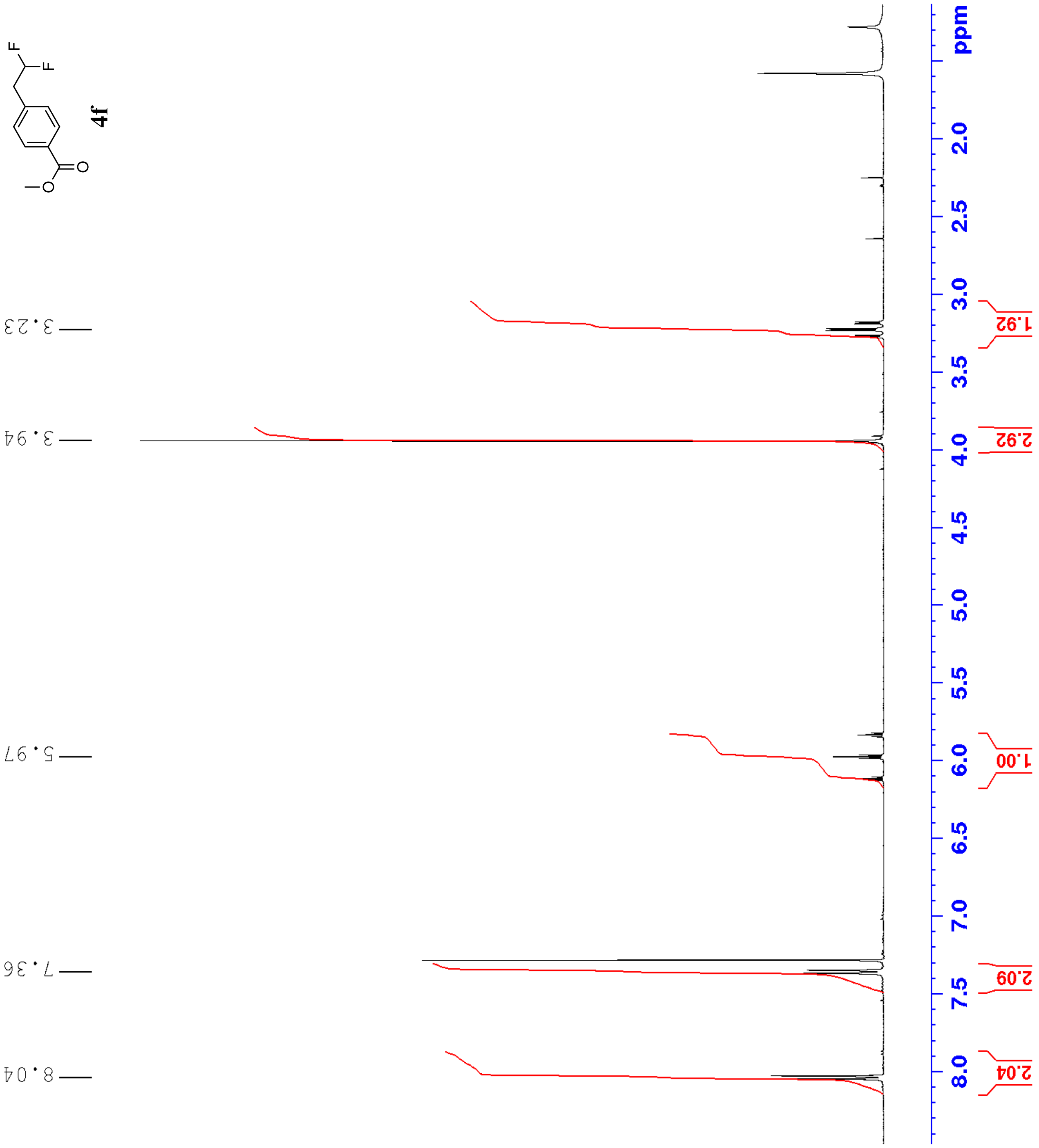

F
F
O
O
8.04
7.36
5.97
3.94
3.23
ppm
2.0
2.5
3.0
3.5
4.0
4.5
5.0
5.5
6.0
6.5
7.0
7.5
8.0
1.92
2.92
1.00
2.09
2.04

4f

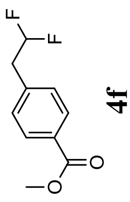

4f

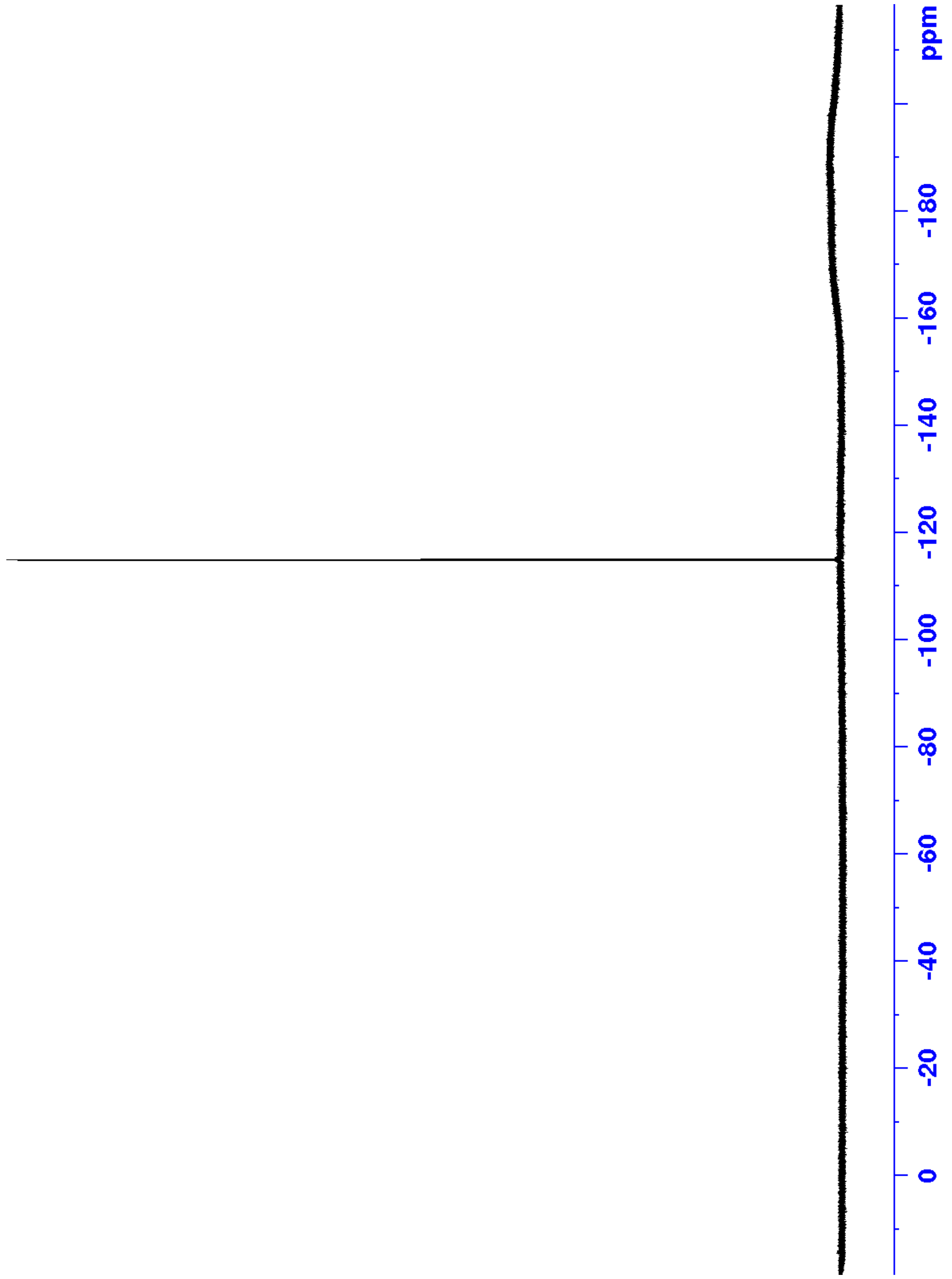

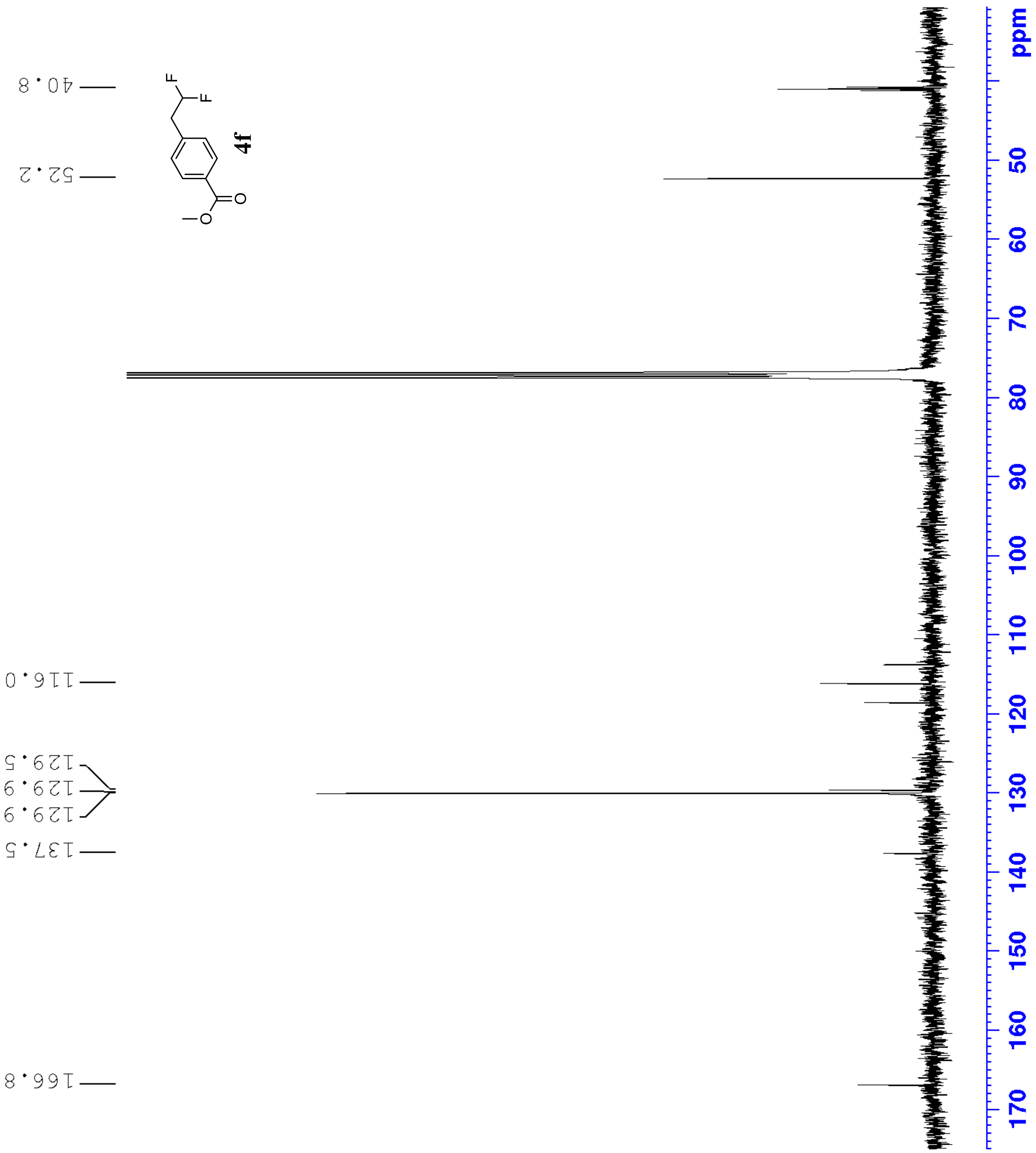
40.8
52.2
116.0
129.5
129.9
129.9
137.5
166.8
4f
ppm
50
60
70
80
90
100
110
120
130
140
150
160
170

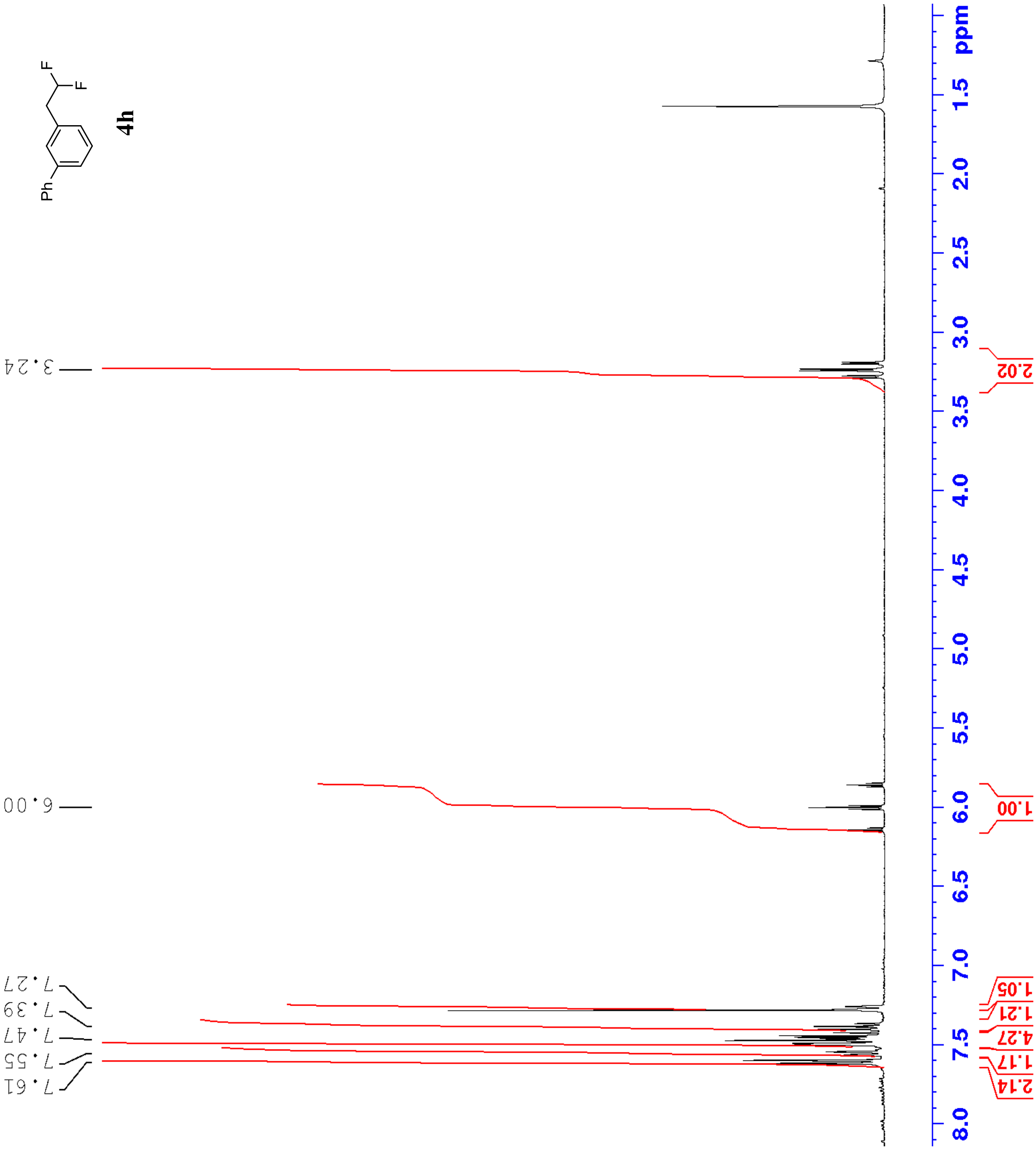

Ph
F
F
4h
3.24
6.00
7.27
7.39
7.47
7.55
7.61
2.02
1.00
1.05
1.21
4.27
1.17
2.14
ppm

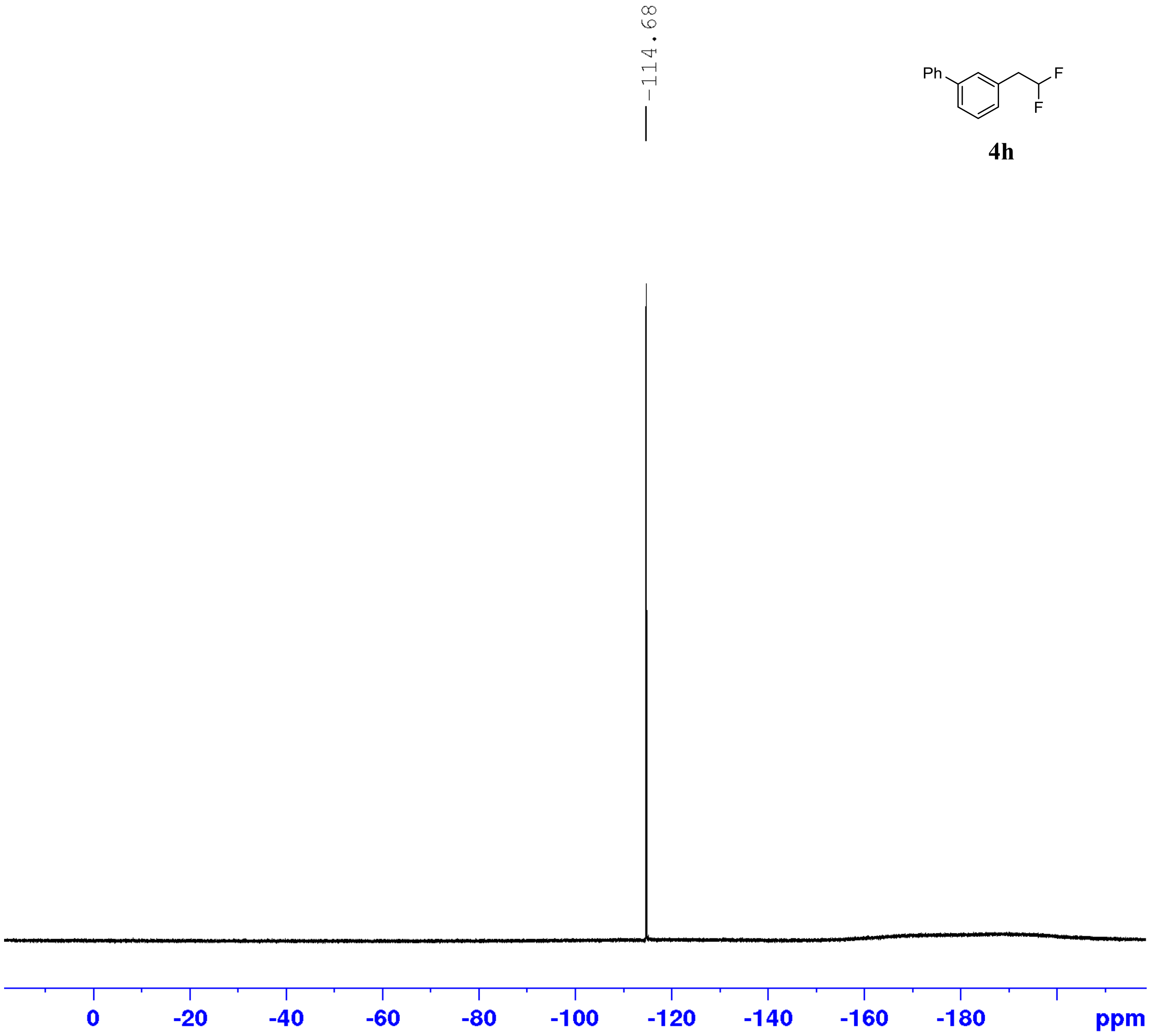
-114.68
Ph
F
F
4h
0
-20
-40
-60
-80
-100
-120
-140
-160
-180
ppm

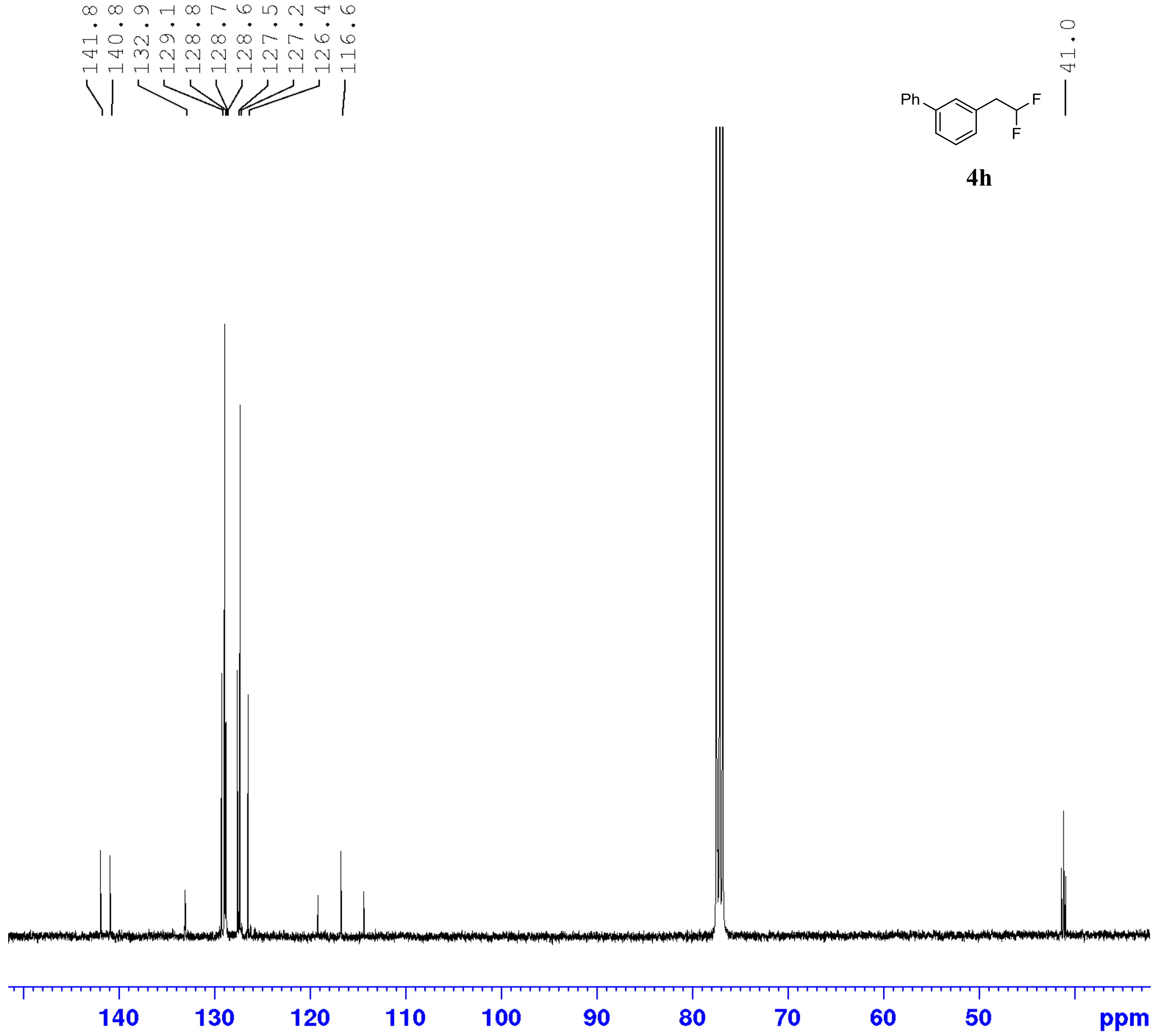

4h
Ph
F
F
41.0
141.8
140.8
132.9
129.1
128.8
128.7
128.6
127.5
127.2
126.4
116.6
140
130
120
110
100
90
80
70
60
50
ppm

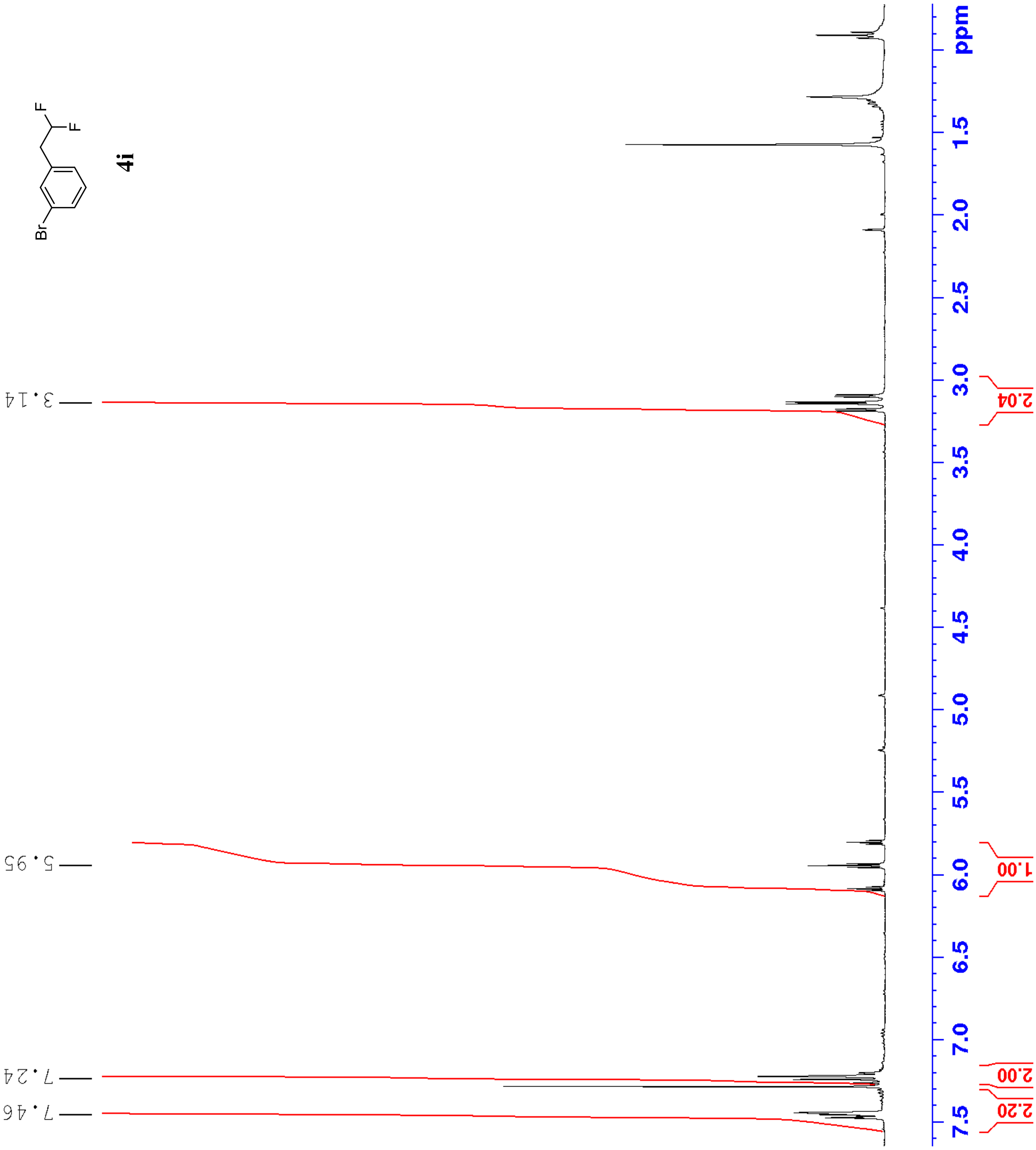
Br
F
F
4i
3.14
5.95
7.24
7.46
ppm
1.5
2.0
2.5
3.0
3.5
4.0
4.5
5.0
5.5
6.0
6.5
7.0
7.5
2.04
1.00
2.00
2.20

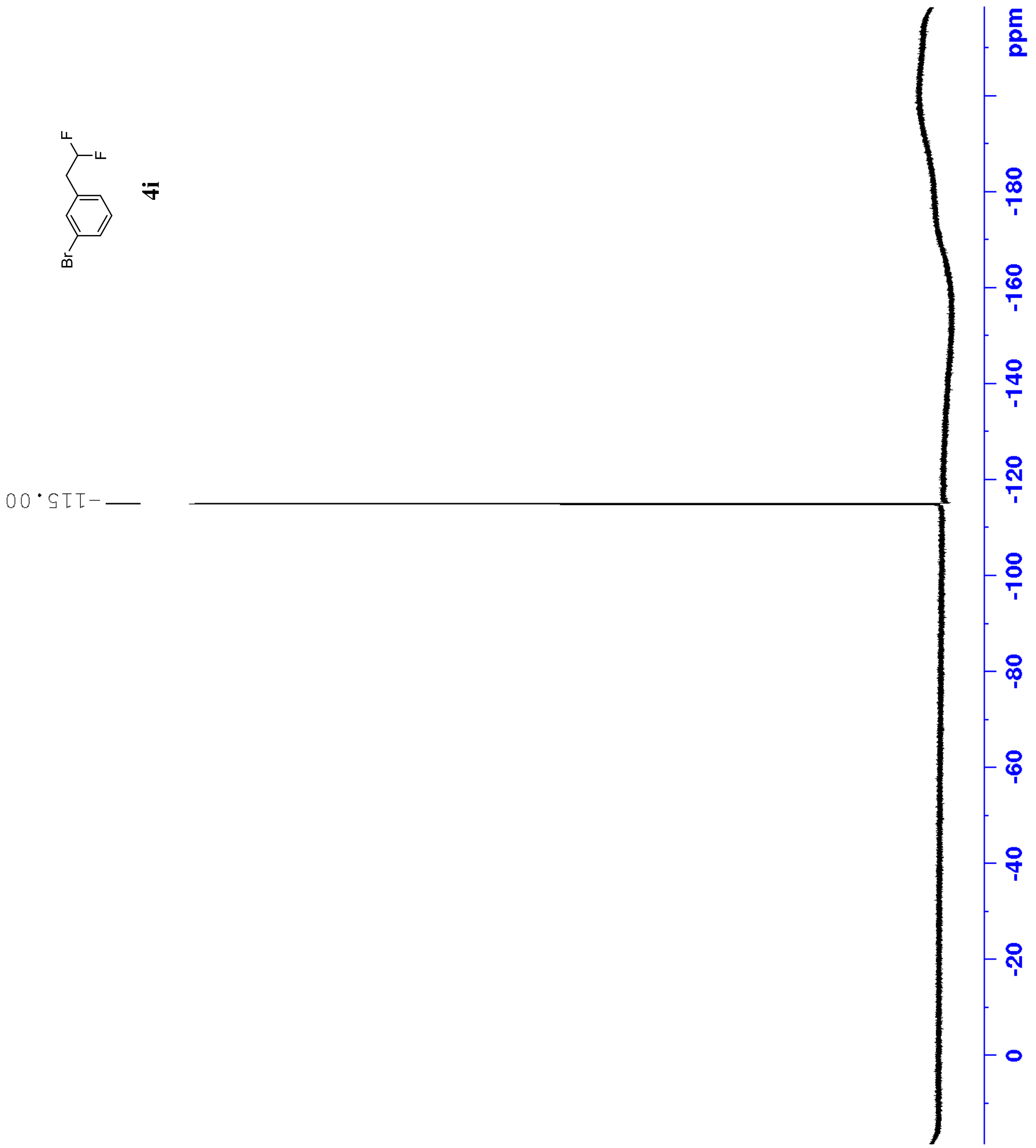
Br
F
F
4i
-115.00
ppm
-180
-160
-140
-120
-100
-80
-60
-40
-20
0

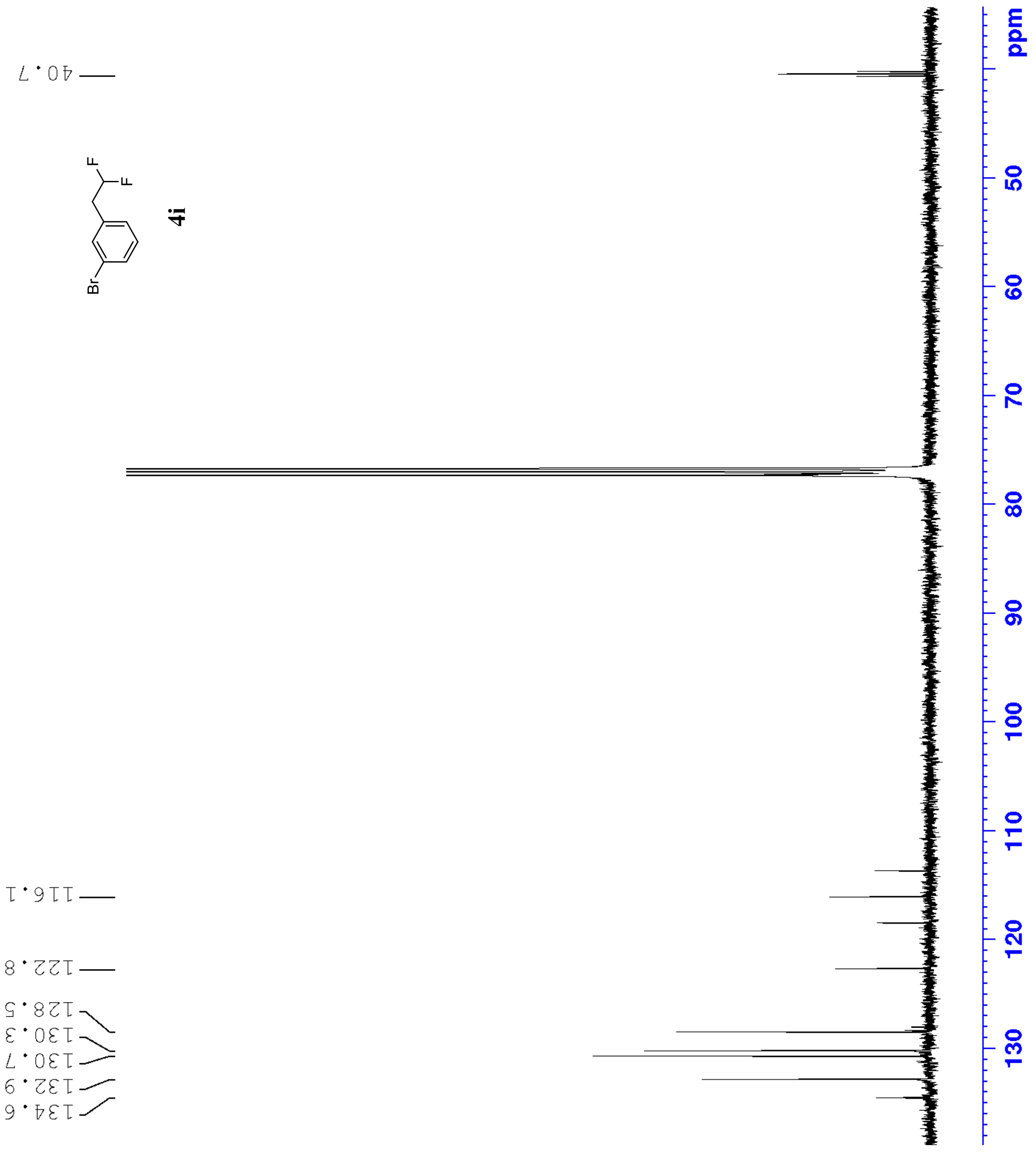
40.7
116.1
122.8
128.5
130.3
130.7
132.9
134.6
Br
F
F
4i
ppm
50
60
70
80
90
100
110
120
130

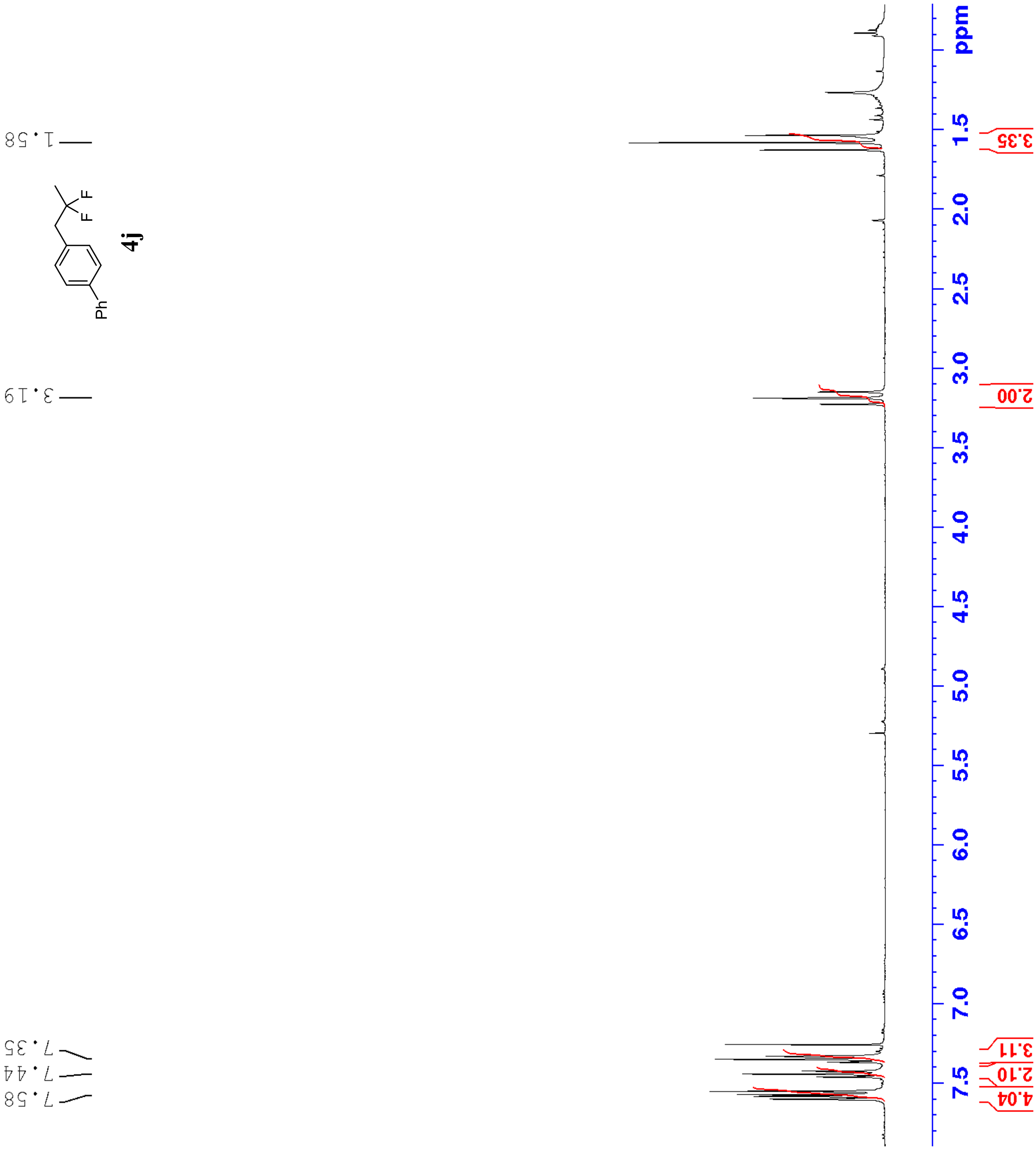
1.58
3.19
7.35
7.44
7.58
Ph
F
F
4j
ppm
1.5
2.0
2.5
3.0
3.5
4.0
4.5
5.0
5.5
6.0
6.5
7.0
7.5
3.35
2.00
3.11
2.10
4.04

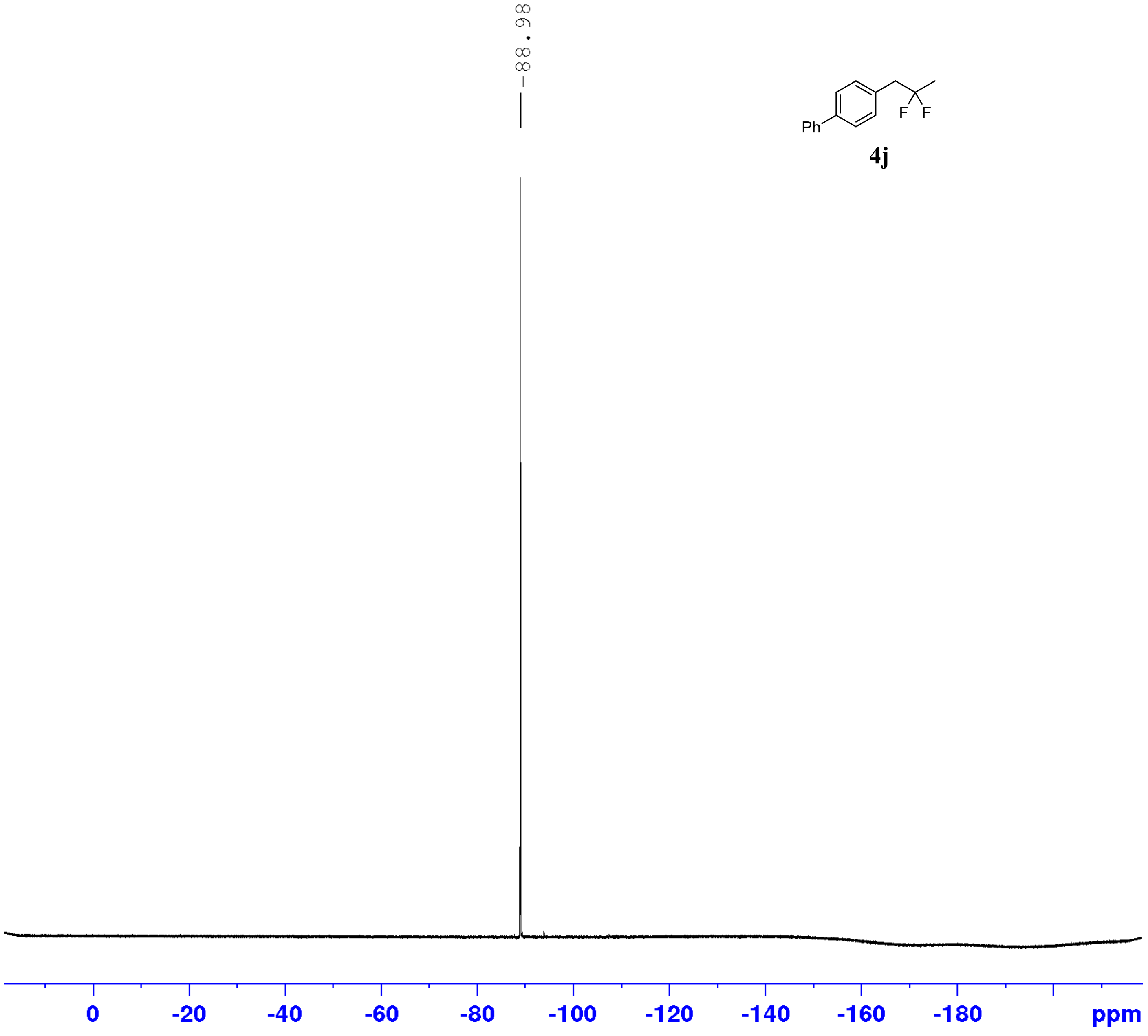
-88.98
Ph
F F
4j
0
-20
-40
-60
-80
-100
-120
-140
-160
-180
ppm

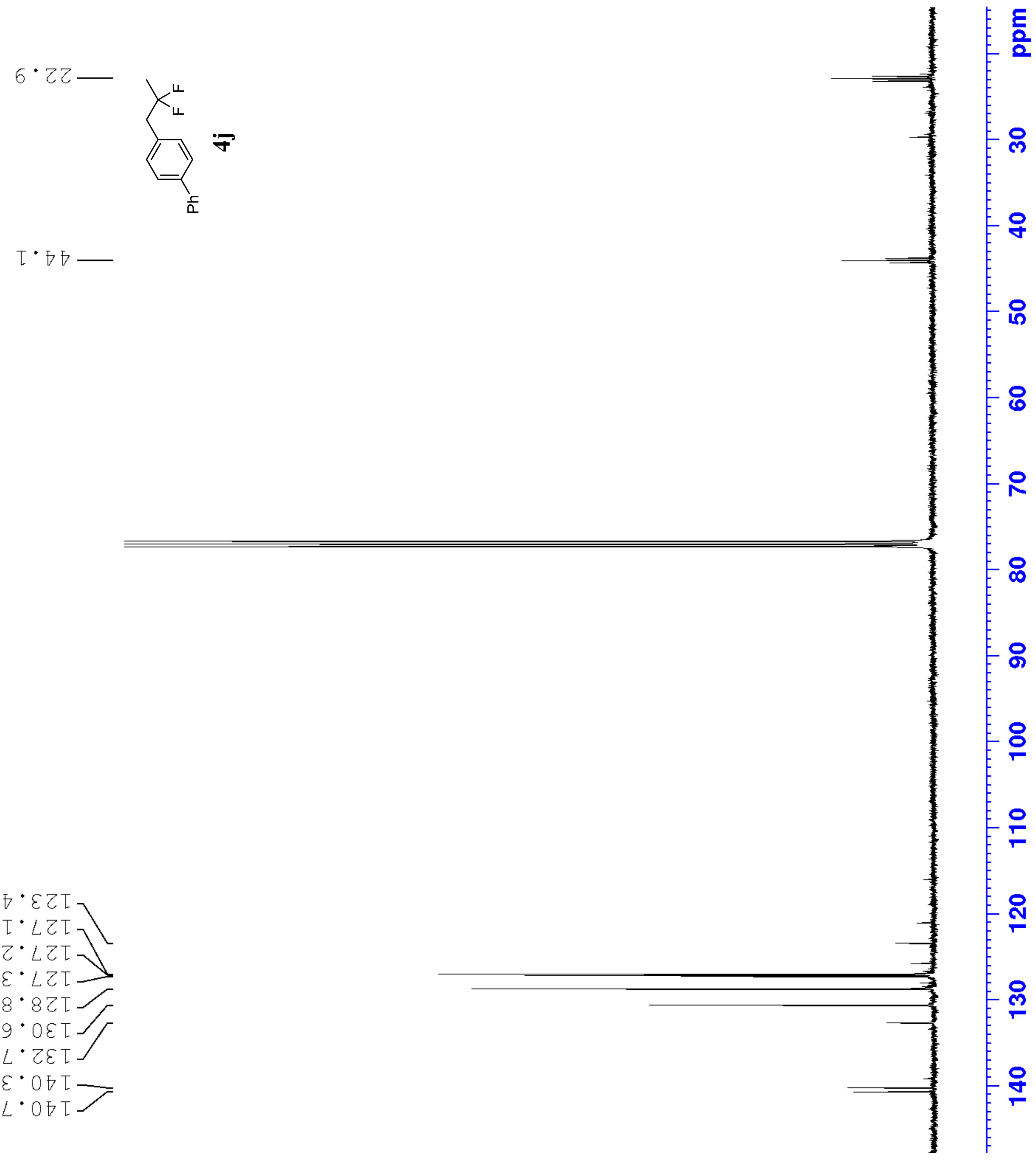
22.9
44.1
123.4
127.1
127.2
127.3
128.8
130.6
132.7
140.3
140.7
Ph
F
F
4j
ppm
30
40
50
60
70
80
90
100
110
120
130
140

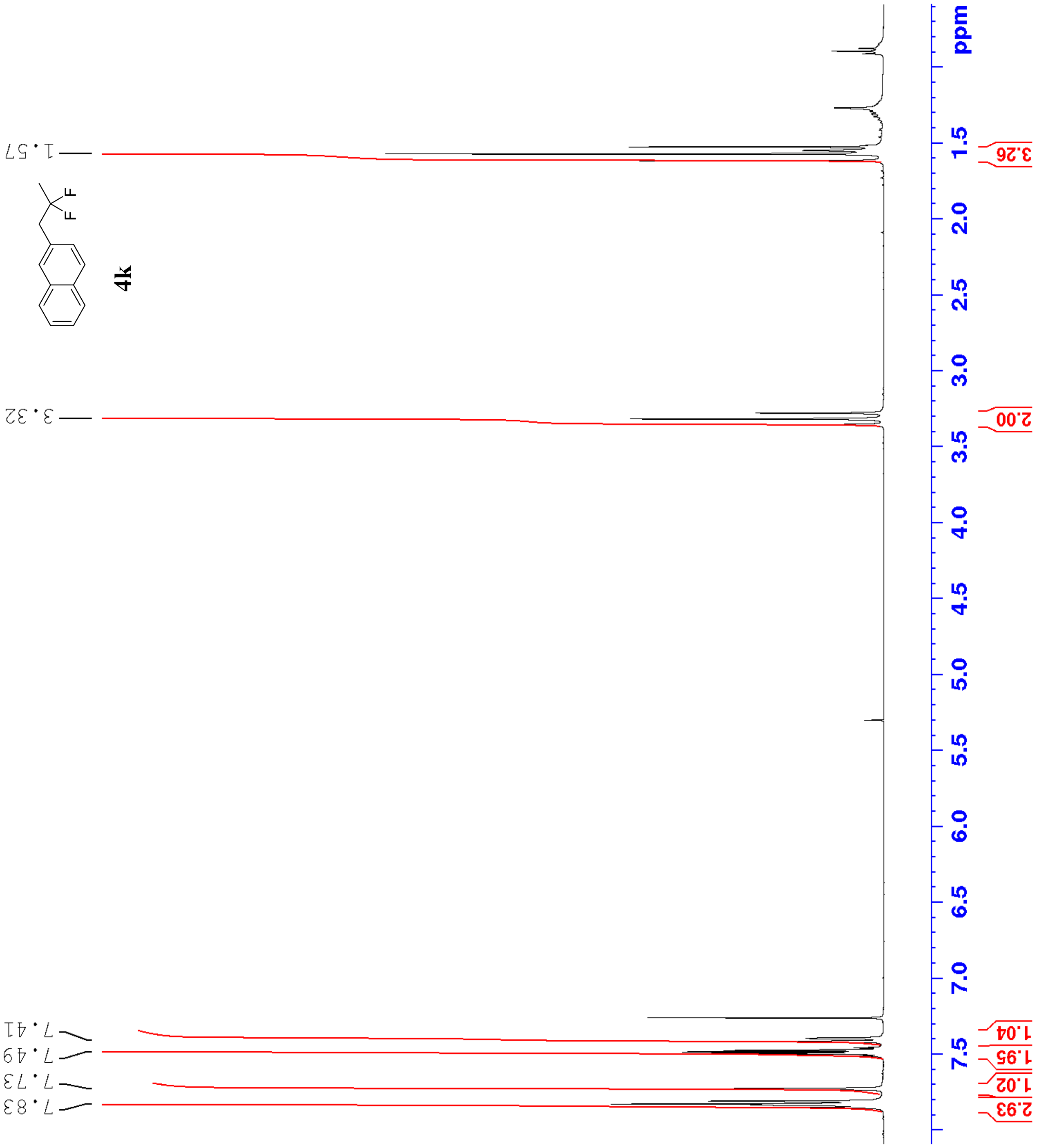

1.57
3.32
7.41
7.49
7.73
7.83
4k
F
F
ppm
1.5
2.0
2.5
3.0
3.5
4.0
4.5
5.0
5.5
6.0
6.5
7.0
7.5
3.26
2.00
1.04
1.95
1.02
2.93

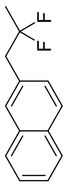

4k

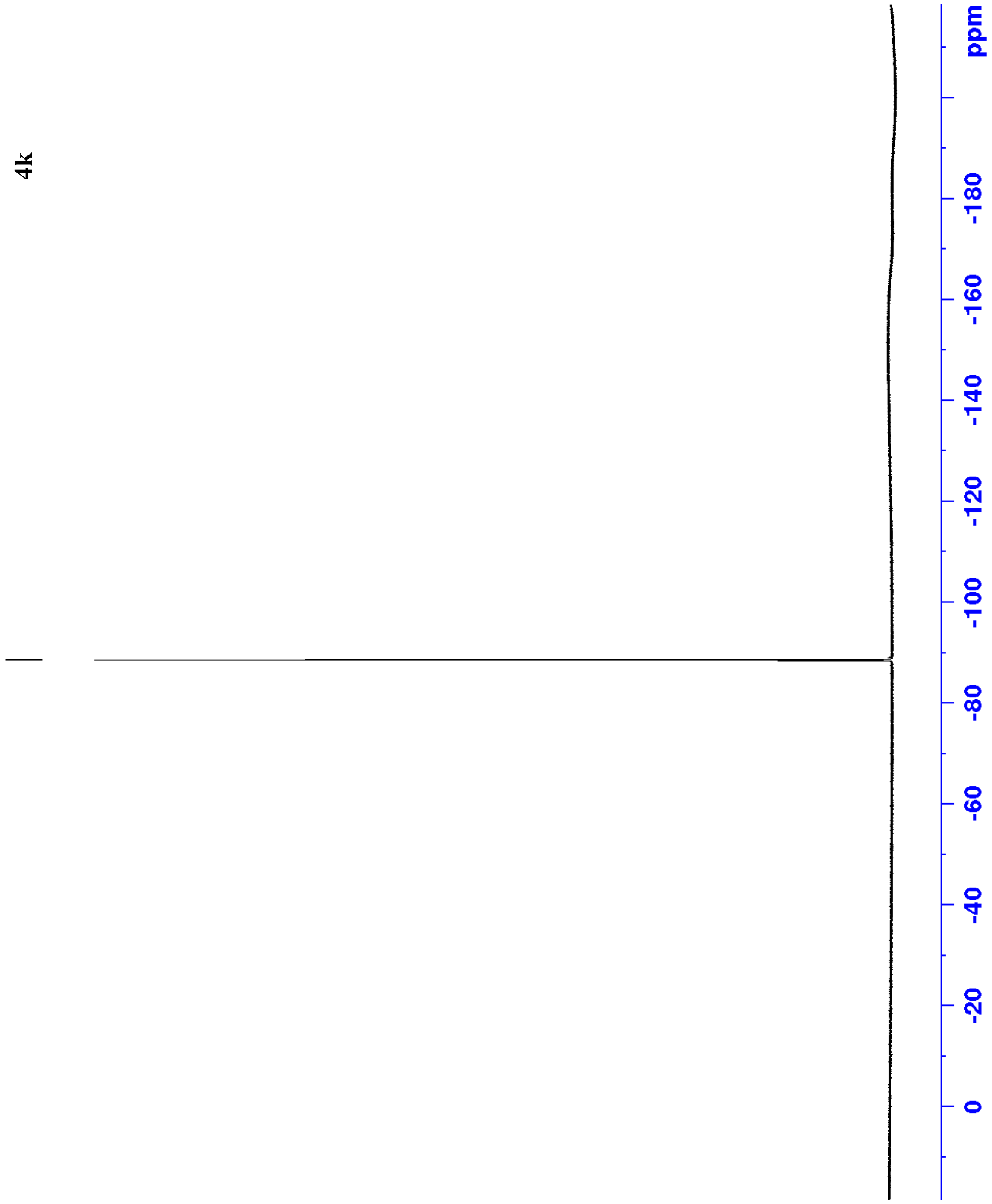

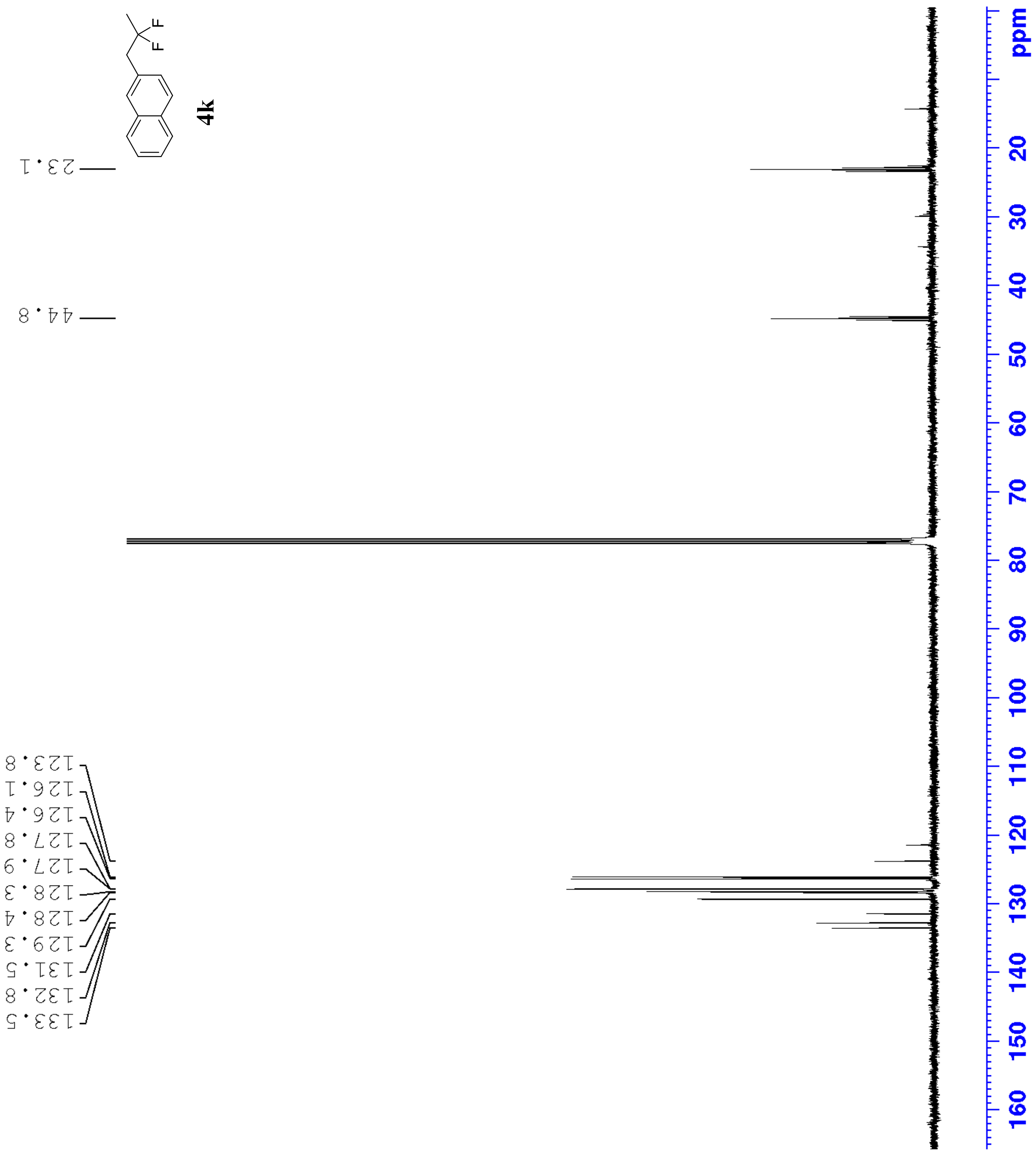
4k
F
F
23.1
44.8
123.8
126.1
126.4
127.8
127.9
128.3
128.4
129.3
131.5
132.8
133.5
ppm
20
30
40
50
60
70
80
90
100
110
120
130
140
150
160

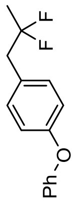

4l

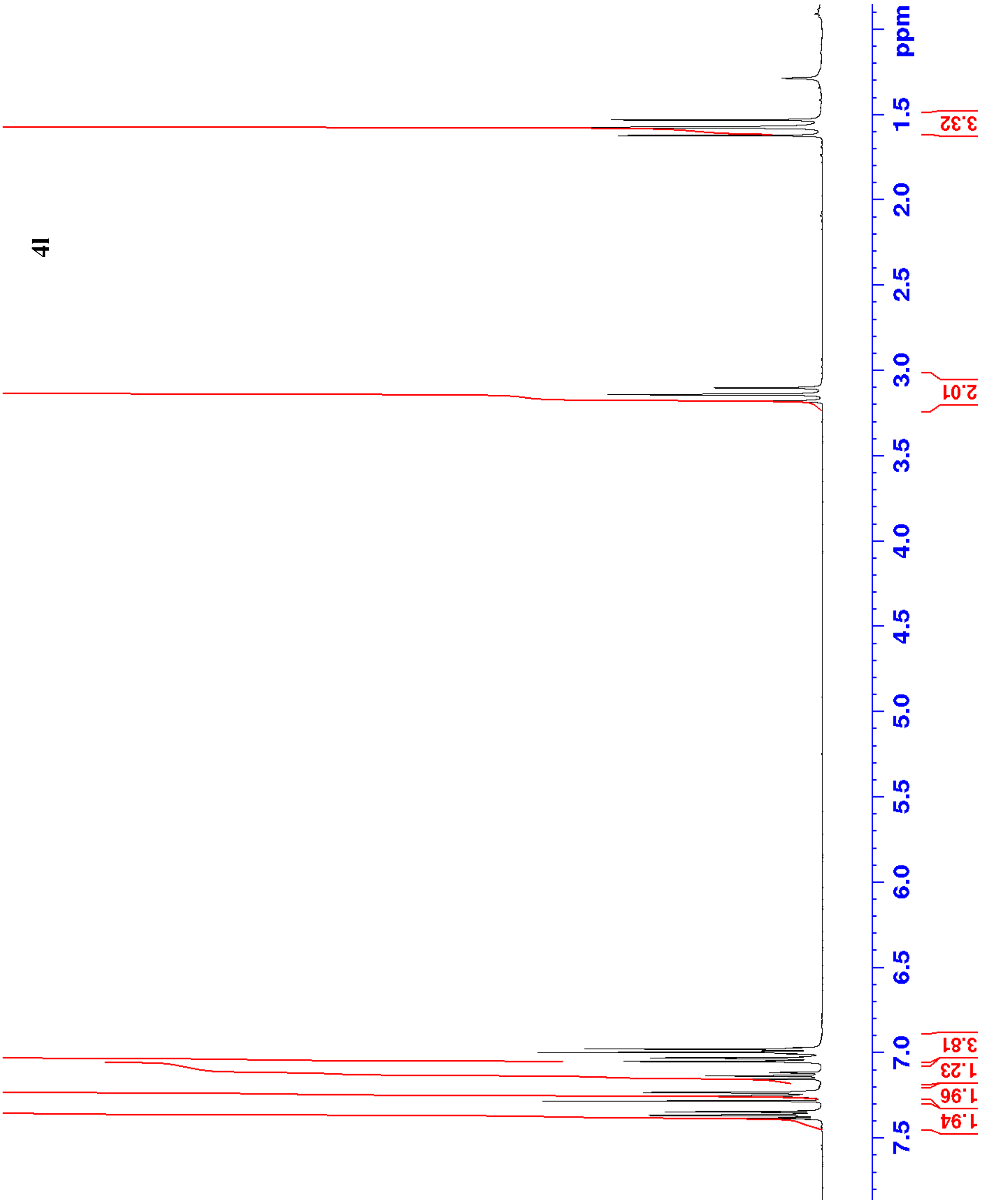
1.58
3.14
7.02
7.14
7.25
7.37
ppm
1.5
2.0
2.5
3.0
3.5
4.0
4.5
5.0
5.5
6.0
6.5
7.0
7.5
3.32
2.01
3.81
1.23
1.96
1.94

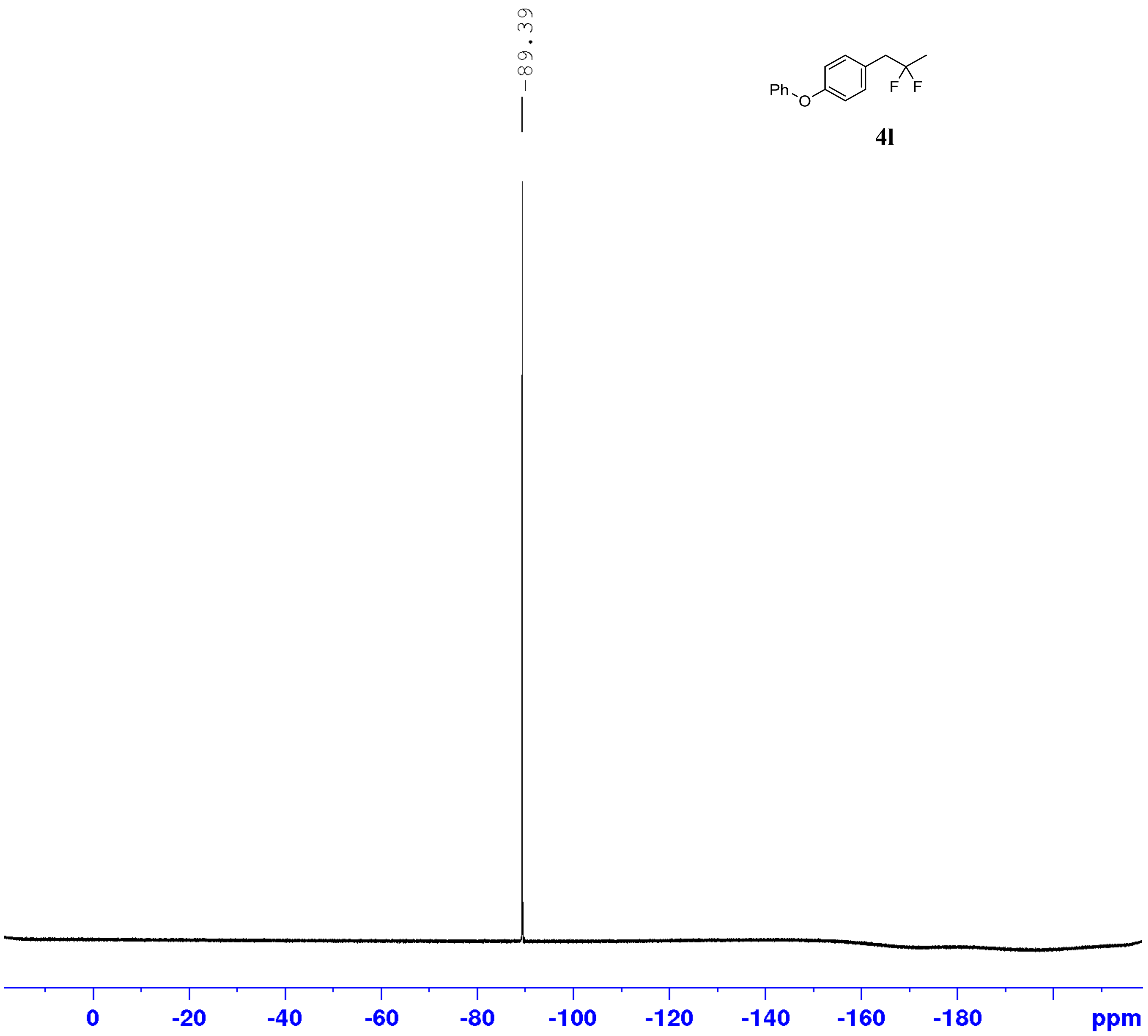
-89.39
Ph
O
F
F
4l
0
-20
-40
-60
-80
-100
-120
-140
-160
-180
ppm

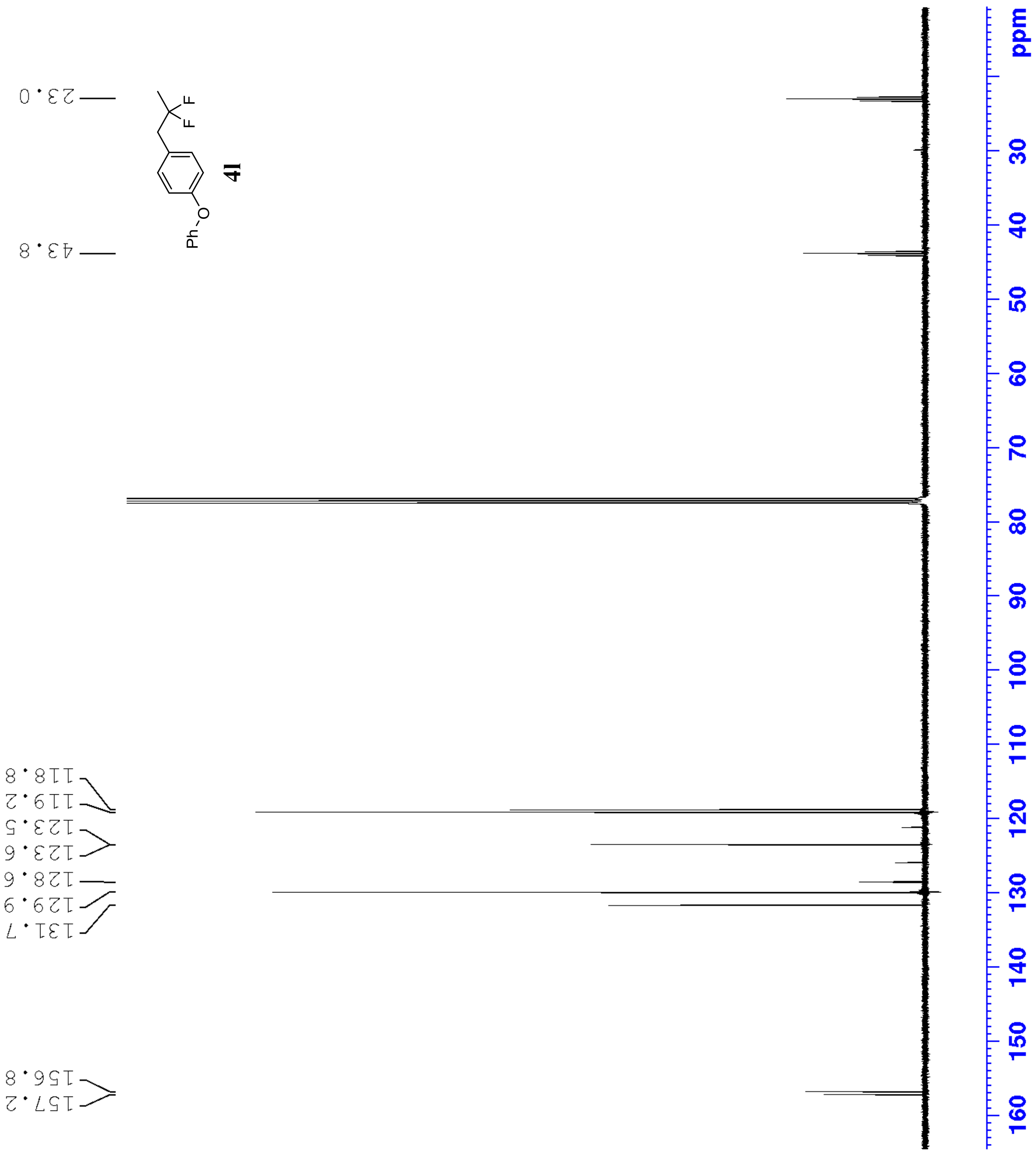
23.0
43.8
118.8
119.2
123.5
123.6
128.6
129.9
131.7
156.8
157.2
Ph
O
F
F
41
ppm
30
40
50
60
70
80
90
100
110
120
130
140
150
160

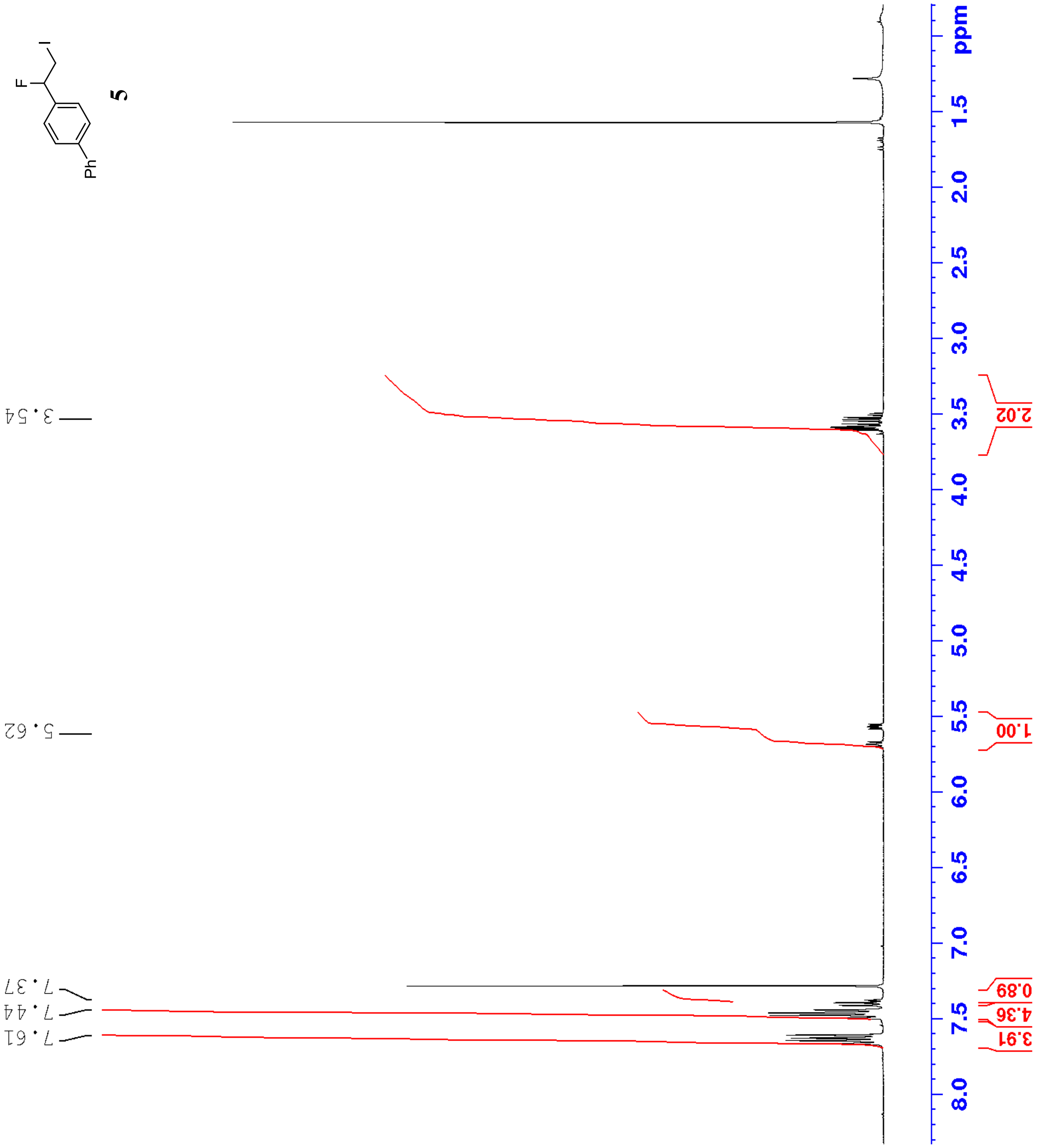
F
I
Ph
5
3.54
5.62
7.37
7.44
7.61
2.02
1.00
0.89
4.36
3.91
ppm
1.5
2.0
2.5
3.0
3.5
4.0
4.5
5.0
5.5
6.0
6.5
7.0
7.5
8.0

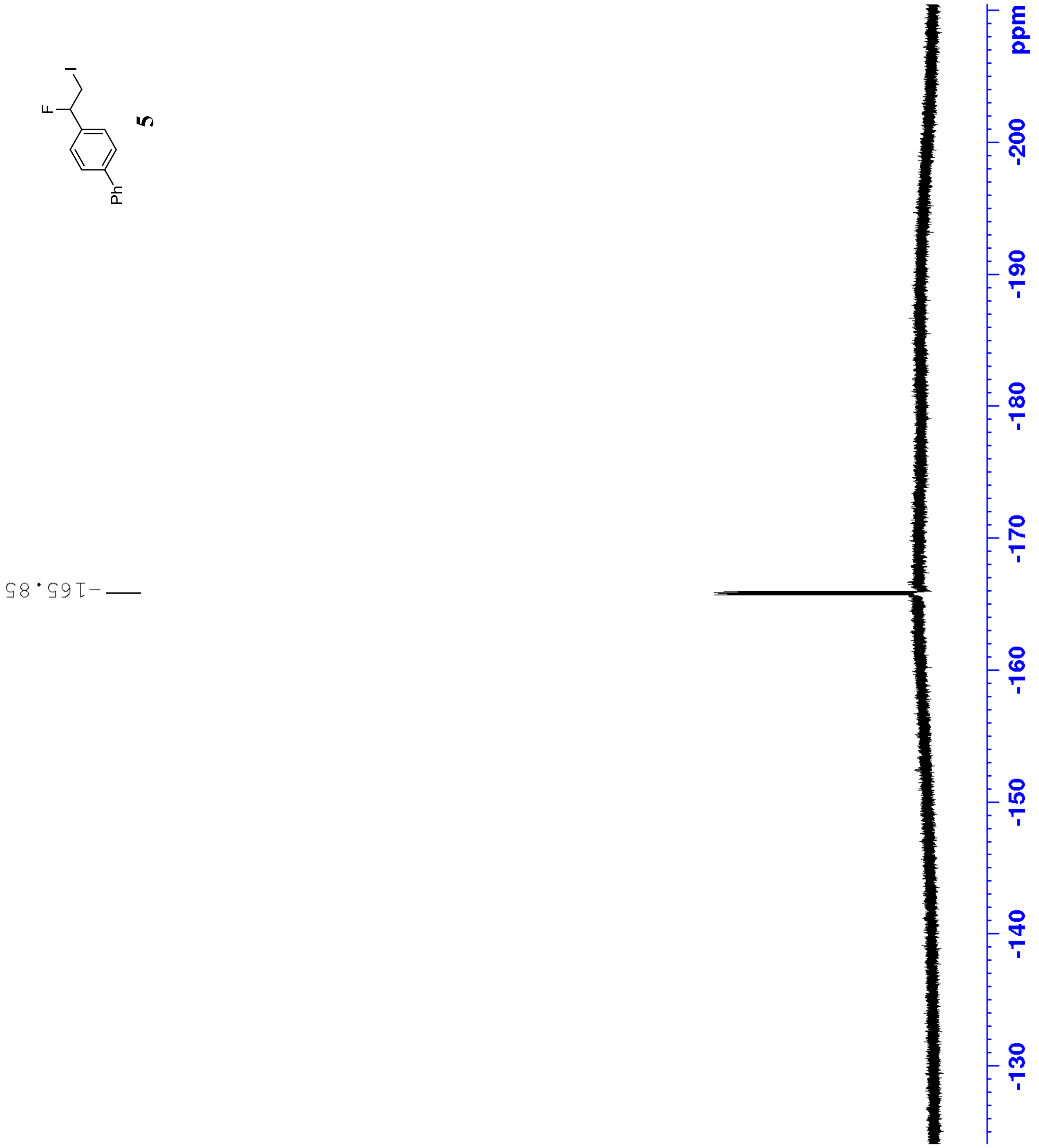

F
I
Ph
5
-165.85
ppm
-200
-190
-180
-170
-160
-150
-140
-130

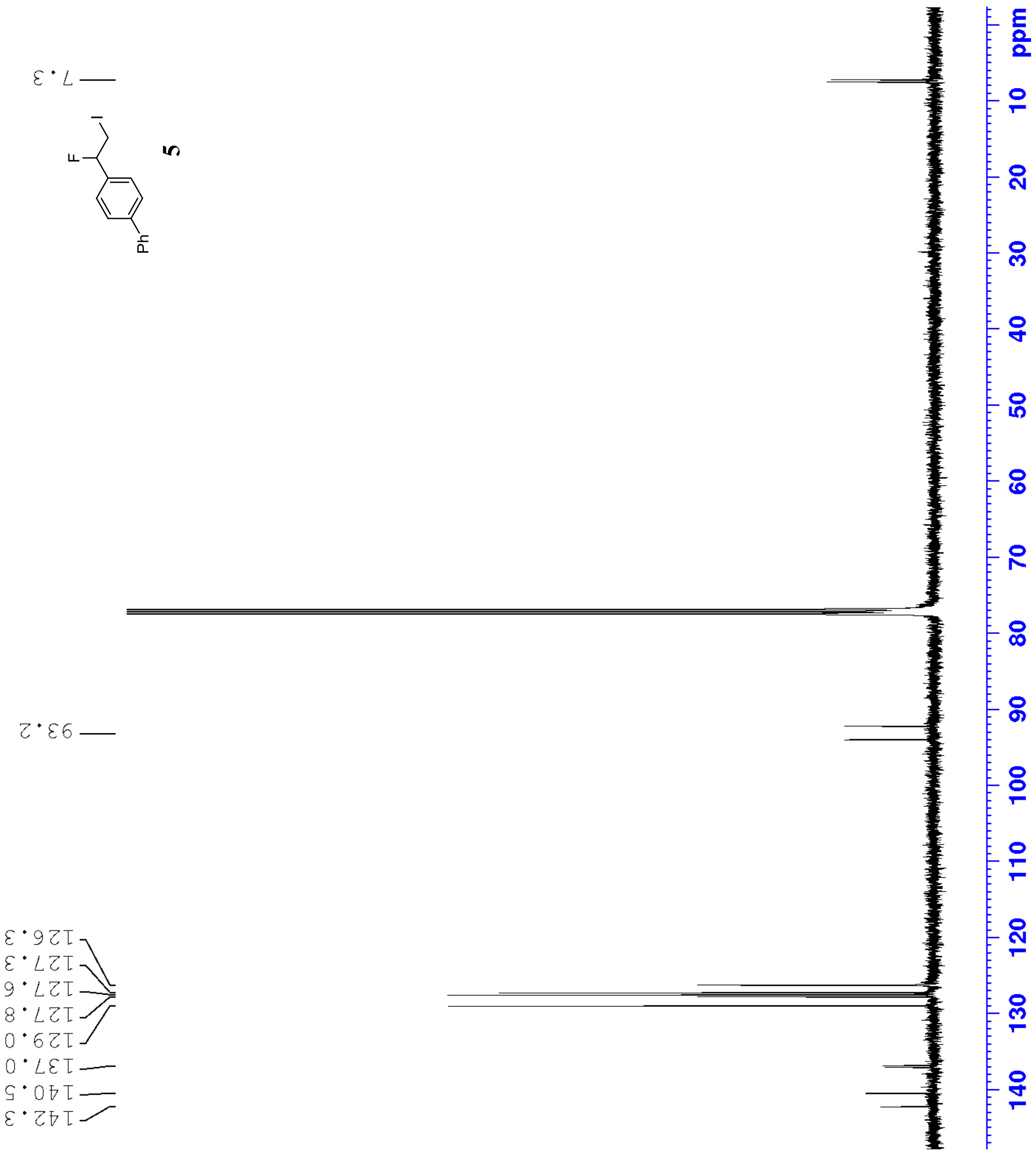
7.3
93.2
126.3
127.3
127.6
127.8
129.0
137.0
140.5
142.3
ppm
Ph
F
I
5
